# NtMYB12 requires for competition between flavonol and (pro)anthocyanin biosynthesis in *Narcissus tazetta* tepals

**DOI:** 10.1186/s43897-023-00050-7

**Published:** 2023-02-08

**Authors:** Jingwen Yang, Xi Wu, Cristina belen Aucapiña, Deyu Zhang, Jiazhi Huang, Ziyuan Hao, Yu Zhang, Yujun Ren, Ying Miao

**Affiliations:** Fujian Provincial Key Laboratory of Plant Functional Biology, Fujian Agriculture and Forestry University, Fuzhou, 350002 China

**Keywords:** NtMYB12, MBW triplex, Flavonol biosynthesis, Proanthocyanin biosynthesis, *Narcissus tazetta*

## Abstract

The color of flowers is one of the main characteristics adopted for plants to attract pollinators to ensure the reproductive success of the plant, they are also important in their ornamental appeal in *Narcissus* plant. In this study, we identified a *NtMYB12* locus encoding an R2R3-MYB transcription factor. Comparative transcriptome analysis of loss- and gain- of *NtMYB12* tissue relative to wild-type narcissus showed NtMYB12 was mainly involved in flavonol and phenylpropanoid metabolic pathways. Biochemical evidences of dual-luciferase activity and chromatin immunoprecipitation assay supported that MYB12 directly bound to promoters of *NtFLS*, *NtLAR,* and *NtDFR* that were cloned by genome walking assay, and activated *NtFLS* and *NtLAR* expression but repressed *NtDFR* expression. More interestingly, NtMYB12 can interact with NtbHLH1 and NtWD40-1 proteins *via* R3 domain that were selected by transcriptome-based WGCNA and confirmed by yeast two hybrid, bimolecular fluorescence complementation and coimmunoprecipitation assay. Interaction of NtMYB12 with NtbHLH1 and NtWD40-1 forming MYB-bHLH-WD40 triplex specially activated *NtDFR* and *NtANS* expression and promoted (pro)anthocyanin accumulation, while NtMYB12 alone activated *NtFLS* and *NtLAR* expression and accumulated flavonols, but repressed *NtDFR* expression. These results indicated that NtMYB12 alone or NtMYB12-bHLH1-WD40-1 triplex requires for competition of metabolism fluxes between flavonol and (pro)anthocyanin biosynthesis.

**Graphical Abstract:**

NtMYB12 dually functions on flavonol and proanthocyanin biogenesis *via* physically binding to *NtFLS* and *NtLAR* promoter activating their expression and on (pro)anthocyanin biosynthesis *via* NtMYB12-NtWD40-NtbHLH (MBW) triplex activating *NtDFR* and *NtANS *expression. Requirement of NtMYB12 alone or MBW complex for the competition between flavonol and anthocyanin biosynthesis results in narcissus colorized petal traits.

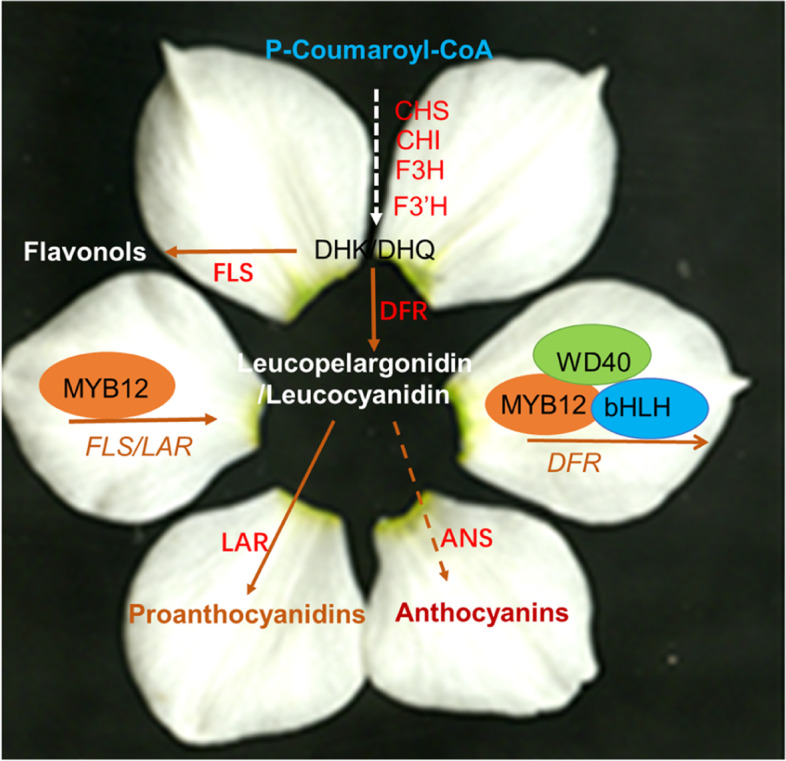

**Supplementary Information:**

The online version contains supplementary material available at 10.1186/s43897-023-00050-7.

## Core

NtMYB12 alone or NtMYB12-bHLH1-WD40-1 triplex requires for competition of metabolism fluxes between flavonol and (pro)anthocyanin biosynthesis in *Narcissus tazetta* tepals to determine colorization of* Narcissus* flower.

## Gene and accession numbers

*NtMYB12* (c106256.graph_c0), *NtFLS* (c101473.graph_c0), *NtLAR* (c28704.graph_c0), *NtANS* (c121090.graph_c1), *NtDFR* (c108471.graph_c1), *NtbHLH1* (c27351/f1p0/1540), *NtWD40-1* (c16261/f1p1/1484).

## Introduction

The color of flowers is one of the main characteristics adopted for plants to attract pollinators to achieve the reproduction of the plant (Amrad et al. 2016; Shang et al. 2011; Peng et al. 2017). Floral color is feature trait determined by a series of metabolites of flavonol/anthocyanin pigments and carotenoids/chlorophyll compounds, which produced from well-known branches of the phenylpropanoid pathway and GSSP carotenoids/chlorophyll pathway (Colquhoun et al. 2011; Colquhoun and Clark 2011; Yamagishi et al. 2014; Ren et al. 2017). Numerous structural and regulatory genes involved in anthocyanin biosynthesis have been functionally addressed and extensively used for the genetic manipulation of floral color (Winkel-Sirley, 2001; Chandler and Tanaka 2007). The regulation of anthocyanin biosynthesis has been shown to act by MYB transcription factors (Koes et al. 2005). Up to now, the activities of these regulators are not restricted to control anthocyanin production, but also have an effect on other metabolite formation such as flavonol, benzenoid, phenylpropanoid or tissue development and stresses tolerance (Colquhoun et al. 2011; Colquhoun and Clark 2011; Schwinn et al. 2006; Zvi et al. 2012; Butelli et al. 2019). For instance, *PhMYB4* locus encodes an R2R3-MYB transcription factor in petunia (*Petunia hybrida*) regulates the expression of Cinnamate-4-hydroxylase (C4H), 4-coumarate CoA ligase (4CL) and FLAVONOL SYNTHASE (FLS), which presumably redirects metabolite flux from anthocyanin biosynthesis to the production of colorless flavonoids, indicating that a low level of *FLS* expression in the petal lobe and a consequent absence of the spatial patterning in *Petunia* flowers (Colquhoun et al. 2011; Colquhoun and Clark 2011; Yamagishi et al. 2014; Yuan et al. 2016).

A link between two metabolites such as benzenoid/phenylpropanoid or flavonol/anthocyanin synthesis pathways can be anticipated due to their biochemical origin, as well as their similar biological role. The diversion of metabolic flux from one branch of phenylpropanoid pathway to another has been reported in *Petunia* (Zuker et al. 2002). For example, the transferring of transcription factor PAP1 (AtMYB75) of *Arabidopsis* into *Petunia* can cause the simultaneous enhancement of both branches of the phenylpropanoid pathway, leading to the production of color and scent in flowers (Zvi et al. 2012). Studies on *Petunia* have revealed a conserved mechanism for the formation of benzenoid/phenylpropanoid components and flavonol/anthocyanin pigmentation in petals. Pigments of anthocyanin are only produced in the overlapping expression domains of the the R2R3-MYB and bHLH coregulators of anthocyanin biosynthetic genes (Schwinn et al. 2006; Albert et al. 2011). Despite the molecular profiles of pigment formation have been addressed in a few plants, the regulatory mechanisms of pigment formation related transcription factors in *Narcissus* plants is limited.

*Narcissus tazetta var* chinensis “Jinzhanyintai” cultivar variety, belongs to the Amaryllidaceous family and is a perennial bulbous plant. It is widely cultivated in East Asia and China with its exquisite flower type and rich fragrance, serving high ornamental value (Remy 2004; Ren et al. 2017). However, its flower color is scarce. A few studies have focused on the identification of single pigment-related gene (Li et al. 2013) and the composition of flavonoids and carotenoids (Li et al. 2015), as well as formation of flavonol (Amwar et al., 2019; Wang et al. 2018). In our previous research, we systematically constructed the pigment metabolic pathways and critical structural genes in *Narcissus tazetta* “Jingzhanyintai” during flower development by transcriptome profiling and pigment metabolite analysis (Ren et al. 2017). By using an individual plant and two similar tissues (tepal and corona) of *Narcissus tazetta* to create an isogenic line, a regulatory network of three branches of the phenylpropanoid pathway of *Narcissus tazetta* was established by using transcriptome-based WGCNA analysis, and highlighted the potential metabolic fluxes of substrate competition in generating patterns of color, and provided several candidate transcription factors including NtMYB12, NtMYB1, NtAP2-ERF, NtbZIP, NtNAC, NtMYB, NtC2C2, NtC2H2, and NtGRAS, which are closely associated with metabolite fluxes by WGCNA analysis (Yang et al. 2021). However, the molecular regulation of the correlation of metabolite components and color pattern is still limited.

In this study, we detailed identified a *NtMYB12* locus encoding an R2R3-MYB transcription factor that regulates the expression of flavonol synthase (*FLS*) and leucoanthocyanidin reductase (*LAR*) genes. Comparative transcriptomic analysis of loss- and gain- of *NtMYB12* tissue relative to wildtype globally addressed NtMYB12 reprogrammed gene expression profile in flavonol and anthocyanin biosynthesis pathway. Biochemical evidence further supportes that MYB12 directly binds to promoter of *NtFLS*, *NtLAR*, dihydroflavonol 4-reductase *NtDFR* and activates *NtFLS* and *NtLAR* expression and represses *NtDFR* expression, leading to form a low level of (pro)anthocyanin and a high level of flavonol in narcissus tepal. By using coexpression network analysis of *NtMYB12*, NtbHLH1 and NtWD40-1 were selected, their interactions were proofed by yeast two hybrid and bimolecular fluorescence assay as well as coimmunoprecipitation assay. Further, NtMYB12 interacting with NtbHLH1 and NtWD40-1 to form MYB-bHLH-WD40 complex specially activated *NtDFR* expression by using in vitro dual-luciferase transient assay and increased anthocyanin accumulation in Arabidopsis, suggesting that NtMYB12 alone or NtMYB12-bHLH1-WD40-1 complex requires for competition between flavonol and (pro)anthocyanin biosynthesis.

## Results

### The NtMYB12 encodes an R2R3-MYB transcription factor highly expressed in the flower

**Fig. 1:**
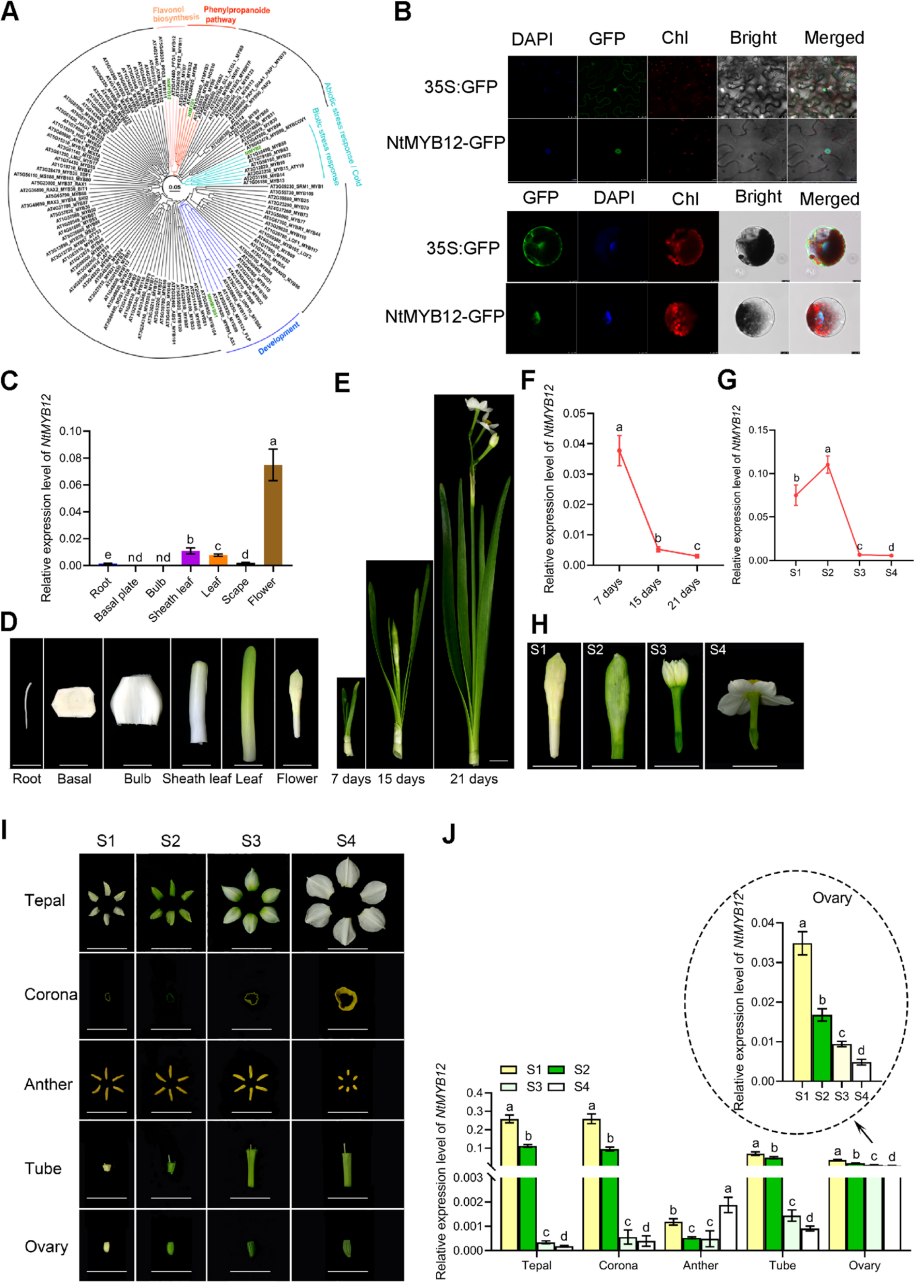
*NtMYB12* expression pattern of various tissues during narcissus development. **A** NtMYB12 is encoded an R2R3-MYB transcription factor by evolutionary analysis; **B** NtMYB12 located in the nucleus by transiently transforming to tobacco leaf epidermal cells and narcissus petal protoplasts. Scale bars = 20 µm; **C-D** *NtMYB12* expression pattern in various tissue. Scale bars = 1 cm. **E–F** *NtMYB12* expressed decreasingly during narcissus plant development; **G-H** *NtMYB12* expressed decreasingly during flower development; **I-J** *NtMYB12* expression pattern in various flower organs. Scale bars = 1 cm. the variance was homogeneous, the new Duncan's multiple range test in one-way ANOVA was used, different letter presents the significant different (*p* < 0.05)

Complete coding sequence of *NtMYB12* contain 819 bases contains the conserved R2R3 domain at the N-terminal end, which is also the DNA binding site of the R2R3 MYB proteins. Three tryptophan residue (W) in R2 domain and one phenylalanine (F) and two tryptophan residue (W) in R3 domain, which is important for formation of the core of helix-turn-helix (HTH) were presented (Yuan et al. 2016). Alignment of NtMYB12 with different R2R3 MYB proteins of Sg7 clade indicated presence of another conserved motif with a consensus sequence SG7 (KRRGGRTSRCTMK) and SG7-2 (WLLE), but there is likely no binding motif of bHLH. MEME software analysis further indicated presence of characteristic signature sequences of the Sg7-2 group of the R2R3 MYB proteins. Apart from C1 and C2 motifs, recently an additional motif known as C3-motif/ZnF-like (zinc finger like) has been identified in the flexible c-terminal of certain activator belonging to R2R3 MYB transcription factors. Similar C3 motif was also located in the C-terminal of NtMYB12 (Supplementary Fig. S1), suggesting its role as a regulator of general flavonol and phenylpropanoid pathway, similar to MdMYB12 related to high content flavonol in red-fleshed apple (Wang et al. 2017). The full length of coding sequence and globally analyzed its evolutionary position in the Fig. 1A.

We further identified NtMYB12 subcellular localization in *Arabidopsis* and *Narcisssus* cells by protoplast transit expression assay, and showed that NtMYB12 fusion GFP was localized in the nucleus and had stronger transcription activity (Fig. 1B). By using RT-qPCR, the transcript abundance profile of *NtMYB12* in different organs was exhibited that *NtMYB12* transcript was lowly expressed in vegetable organ (e.g., root, bulb, leaf, scarp), while it highly expressed in the reproductive organ tepals and corona (Fig. 1C-D); During the period of narcissus plant development from 7 to 21 days, the *NtMYB12* expression level was declined especially 10 folds from stage 1 to stage 4 of flower development (Fig. 1E-F), *NtMYB12* expression level declined significantly in tepal, corona, tube, and ovary, but increased at stage 4 in anther (Fig. 1G-I), which accompanied with these tissues were colorless, fait yellow, green and white colorization. It seems that this gene is closed related to the color formation in *Narcissus tazetta* “Jinzhanyintai” tepals.

### Loss- /gain- of NtMYB12 alters flavonol and anthocyanin biosynthesis related genes expression

**Fig. 2:**
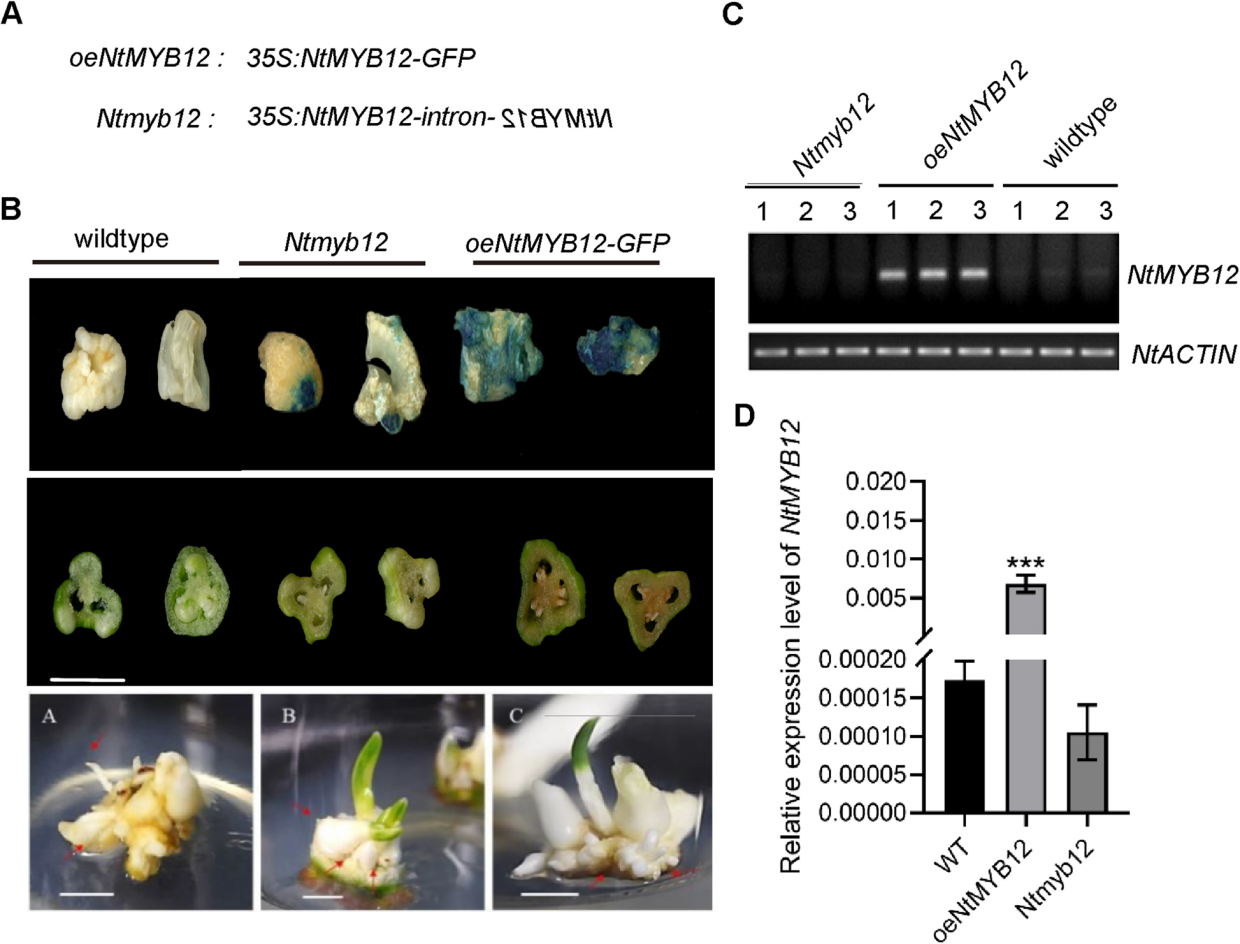
Gain/Loss of NtMYB12 transgenic callus was generated and identified. **A** Scheme of gain/loss of *NtMYB12* constructs; **B** overexpressing *NtMYB12* and *NtMYB12* RNAi transgenic bulbs (*Ntmyb12*) were generated; Scale bars = 1 cm; **C** Identification of transgenic tissues by semi-RT-qPCR, *NtACTIN* gene was set up as an inner control; **D** Identification of transgenic bulbs by RT-qPCR. The error bar presented standard deviants of three biological replicates. Asterisk presents the statistical significance compared to WT (* *p* < 0.05; ** *p* < 0.01; ***p < 0.001)

In order to illustrate NtMYB12 function, loss- of *NtMYB12* (*NtMYB12-RNAi*, *Ntmyb12*) and gain- of *NtMYB12* (*oeNtMYB12*) constructs were generated. Callus cells of narcissus transformed with the mentioned construct (Fig. 2A) resulted in differentiation of callus firstly in differentiation medium supplemented with kanamycin (25 mg/l), which was detected positive transforms by GUS staining, then emergence of putative embryos on embryo development medium. The embryos appeared globular and lead to generation of white translucent secondary embryos. Emerging embryos converted to small bulb shoots on shoot development medium. Individual bulb shoots were numbered and then multiplied on shoot multiplication medium. Rooting of putatively transformed shoots was carried out on rooting medium and later transplanted in the green house (Patent CN202210162166.8) (Fig. 2B). Reverse transcript semi-quantitative PCR and RT-qPCR were used to proof *Ntmyb12* knockdown and overexpressing *NtMYB12* transforms. The transcript level of *Ntmyb12* could not detect in the gel by semi-RT-qPCR, while stronger signal appeared in *oeNtMYB12* and weaker signal appeared in wildtype. *NtACTIN* was used as an inner control (Fig. 2C-D).

**Fig. 3:**
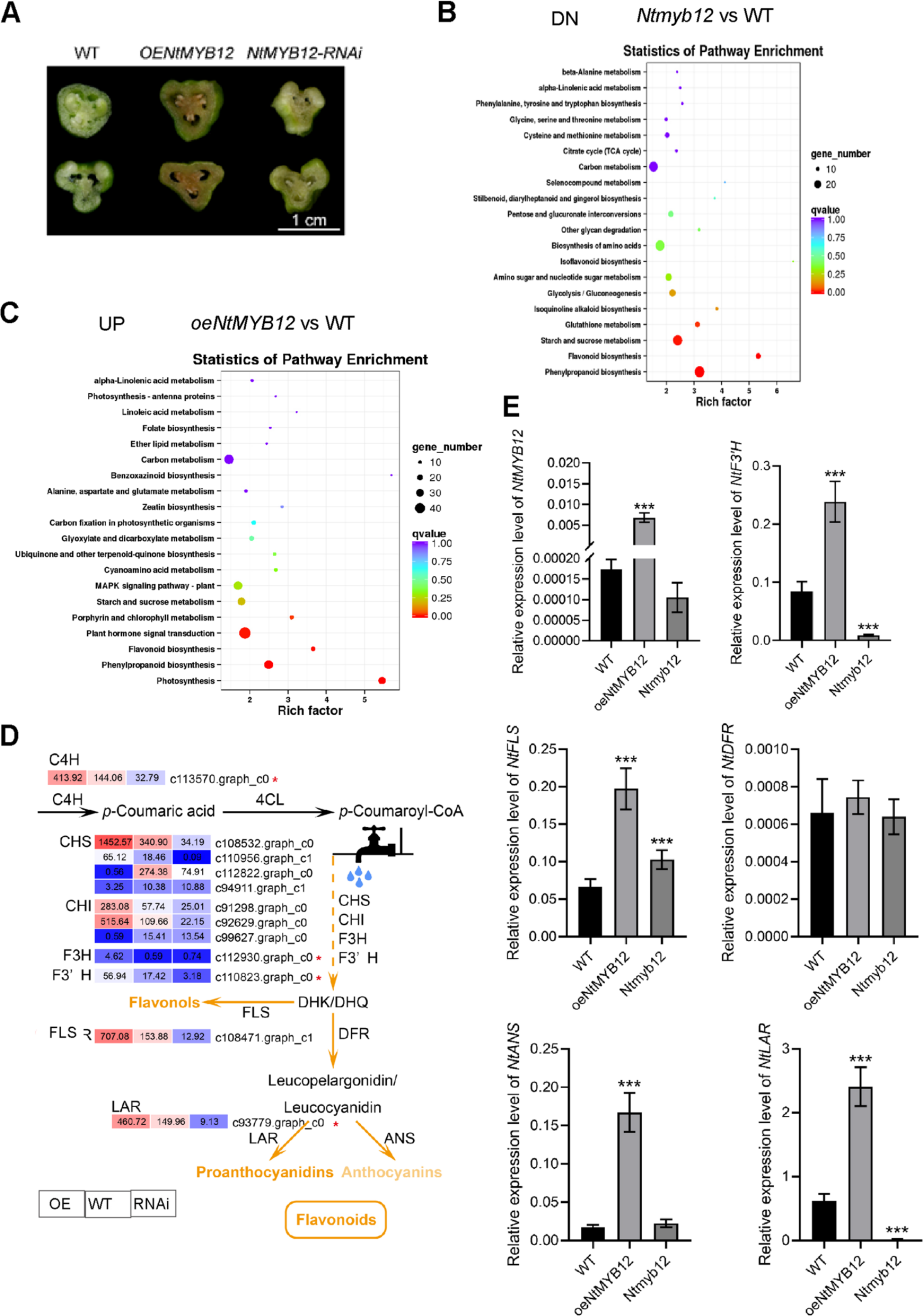
The KEGG pathways of DEGs of *Ntmyb12* or *oeNtMYB12* relative to WT (IFCI >  = 2, *P* value < 0.01) were enriched in phenylpropanoid biosynthesis and flavonoid biosynthesis pathways. **A** The tissues used for RNA-seq; **B** Down-regulated enrichment pathways of DEGs from *Ntmyb12* relative to WT by KEGG analysis; **C** Up-regulated enrichment pathway of DEGs from overexpressing *NtMYB12* line relative to WT by KEGG analysis; **D** The heatmap of DEGs mapped in the flavonol and anthocyanin biosynthesis pathways; **E** The expression levels of *NtFLS, NtLAR, NtF3H, NtDFR,* and *NtANS* were indicated by RT-qPCR. The error bar presented standard deviants of three biological replicates (*n* = 9). Asterisk presents the statistical significance compared to WT (* *p* < 0.05; ** *p* < 0.01; ***p < 0.001)

To investigate the function of NtMYB12 on downstream nuclear genetic reprogramming, gain- of function or loss- of function of *NtMYB12* tissues (Fig. 3A) were collected to perform genome-wide transcriptome sequencing. Total 26,480 unigenes (> 1 kb) were collected. Three replicates were got low variant (r2 = 0.97) (Supplementary Fig. S2). Comparison of *oeNtMYB12* or *Ntmyb12* reprograming transcriptome relative to wild-type plants enriched 497 DEGs (ICFI >  = 2, *P* value < 0.01) with 340 transcripts upregulated and 157 transcripts downregulated (Supplemental Dataset S1), or 876 DEGs (ICFI >  = 2, *P* value < 0.01) with 320 transcripts upregulated and 357 transcripts downregulated, respectively (Supplementary Dataset S2). The DEGs (ICFI >  = 2, *P* value < 0.01) of *Ntmyb12* relative to WT were subjected to MapMan analysis (Thimm et al., 2004). The biological processes with the most significant alteration were identified that protein metabolic process and cellular process particularly, protein catalytic activity and protein binding were the most significant terms, closely followed by biological regulation and response to stimulus, particularly, protein transporter activity, then was followed by localization and cellular component organization or biogenesis (Supplementary Fig. S2A), which did not surprisingly consider that metabolite biogenesis was affected by NtMYB12, similar to the closet homologous of MdMYB12 related to high content flavonol in red-fleshed apple (Wang et al. 2017).

To address the DEGs enrichment pathway of biological process and molecular function, these DEGs were further analyzed using KEGG pathway. Expectedly, the most significant downregulated pathway was photosynthesis, phenylpropanoid biosynthesis, flavonoid biosynthesis pathways; the second significant down-enrichment pathway was plant hormone signal transduction pathway and porphyrin and chlorophyll metabolism pathways in the *Ntmyb12* line relative to WT (Fig. 3B; Figure S2B). In contrast, the most significant upregulated pathway was photosynthesis, phenylpropanoid biosynthesis, flavonoid biosynthesis pathways, the second significant enrichment pathway was plant hormone signal transduction pathway and porphyrin and chlorophyll metabolism pathways in the *oeMYB12* line relative to WT (Fig. 3C; Figure S2C).

To characterize up- and downregulated genes of phenylpropanoid biosynthesis and flavonoid biosynthesis pathways in the *Ntmyb12* line (Fig. 3A), these two subsets of genes were selected and analyzed using heatmap and MapMan metabolite pathway matching (Fig. 3B). Intriguingly, *4CL* (c107941; c113409), *C4H* (c113570), *CHS* (c108532), *CHI* (c92629), *FLS* (c108471), *LAR* (c93779), and *CCoMAOT* (c91328) locus encoding enzymes of flavonoid biosynthesis pathway were significantly upregulated in *oeNtMYB12* line, but downregulated in *Ntmyb12* line. However, *4CL* (c113374), *AOC3* (c102005), *BPBT* (c92663), *HCT* (c95028), *CCoMAOT* (c96475) locus encoding enzymes of phenylpropanoid biosynthesis pathway were downregulated in *Ntmyb12* line, and upregulated in *oeNtMYB12* line (Fig. 3D, Supplementary Fig. S3). In order to focus on the pigment formation related flavonol and anthocyanin pathway, the RT-qPCR further confirmed the alteration of transcript level of the selected genes in *oeNtMYB12* and *Ntmyb12* mutant lines relative to the wild-type (Fig. 3E). Gain- or loss- of *NtMYB12* up- or down-regulated *NtF3’H*, *NtFLS*, *NtLAR* gene expression, overexpressing *NtMYB12* also upregulated *NtANS* gene expression in contract, knockdown *Ntmyb12* downregulated their expression*,* however*,* knockdown *Ntmyb12* did not detect *NtANS* transcript level, the transcript level of *NtDFR* was extremely low and could not detect its alteration in overexpressing *NtMYB12* or *Ntmyb12* callus (Fig. 3E). We further detected the transcript level of *NtANS* and *NtDFR* from transiently transformed narcissus petal protoplasts. The transcript level of *NtANS* was significantly upregulated in transformed *oeNtMYB12* petal protoplasts and downregulated in transformed *Ntmyb12* petal protoplasts compared to transformed empty vector petal protoplasts. In contract, the transcript level of *NtDFR* were significantly downregulated in transformed *oeNtMYB12* petal protoplast and upregulated in transformed *Ntmyb12* petal protoplasts, compared to transformed empty vector petal protoplasts (Supplementary Figure S4). These results suggest that NtMYB12 was involved in regulating flavonoid and proanthocyanin biosynthesis pathways.

### NtMYB12 directly targets and regulates *FLS, DFR, *and *LAR* gene expression in flavonoid biosynthesis

**Fig. 4:**
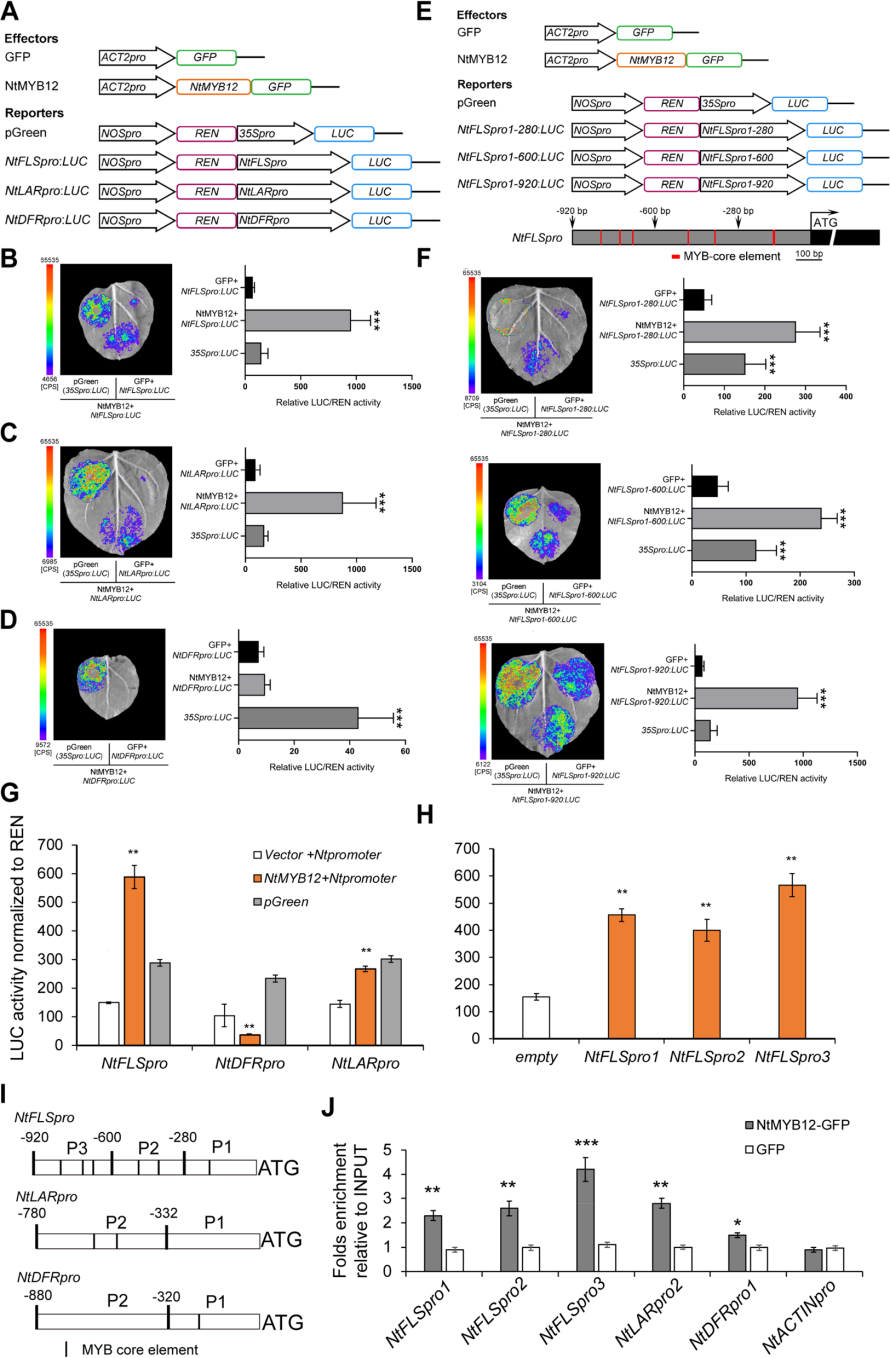
NtMYB12 directly regulates *NtFLS*, *NtLAR* and *NtDFR* gene expression. **A** Scheme of constructs in dual luciferase activity assay; **B** luciferase fluoresce imaging and luciferase activity of cotransformed tobacco leaves with *oeNtMYB12* and *NtFLSpro:LUC* relative to empty vector with *NtFLSpro:LUC;* **C-D** luciferase fluoresce imaging and luciferase activity of cotransformed tobacco leaves with *oeNtMYB12* and *NtDFRpro:LUC* (C)or *NtLARpro:LUC* (D) relative to empty vector with *NtFLSpro:LUC;* **E** Scheme of constructs of various fragment *NtFLSpro:LUC* in dual luciferase activity assay; **F** luciferase fluoresce imaging and luciferase activity of cotransformed tobacco leaves with *oeNtMYB12* and various fragment *NtFLSpro:LUC* relative to empty vector with *NtFLSpro:LUC;* **G** luciferase activity of cotransformed *oeNtMYB12* with *NtFLSpro:LUC* or *NtDFRpro:LUC* or *NtLARpro:LUC* relative to empty vector with *Pro:LUC* in narcissus tepal protoplast; **H** luciferase activity of cotransformed oeNtMYB12 with various fragment *NtFLSpro:LUC* relative to empty vector with *NtFLSpro:LUC* in narcissus tepal protoplast; **I** Scheme of various promoter fragments of *NtFLSpro*, *NtLARpro* and *NtDFRpro* in ChIP-qPCR assay (**J**) Fold change enrichment normalized to INPUT of *oeNtMYB12* on the promoters of *NtFLS, NtDFR, NtLAR* by ChIP. *NtACTIN* was used as an inner control, GFP alone was used as IP control. The error bar presented standard deviants of three biological replicates (*n* = 9). Asterisk presents the statistical significance compared to GFP plant (* *p* < 0.05; ** *p* < 0.01; ***p < 0.001)

The previous work showed *NtMYB12* coexpression with *NtFLS* and *NtLAR* (Yang et al. 2021). With the petal development from stage 1 to stage 4, petal color accompanies with colorless, yellow, fain yellow, then white, the color pigment flavonoids (Lutin and Maringen) content were firstly up accumulated at stage 1, then went down from stage 2 to stage 4 (Ren et al. 2017; Yang et al. 2021), thus, NtMYB12 might directly regulates *NtFLS*, *NtDFR, NtLAR,* and *NtANS* gene expression in flavonol and (pro)anthocyanin biosynthesis. In order to clarify whether NtMYB12 physically regulated *NtFLS*, *NtDFR, NtLAR,* and *NtANS* gene expression, the promoters of *NtFLS, NtDFR,* and *NtLAR* gene were successfully cloned and registered in database by using genome walking assay, since the genome sequence of *Narcissus* was not created up to now. Expectedly, the promoter sequences of *NtFLS, NtLAR,* and *NtDFR* contain many MYB-motives (Table S2). Dual luciferase report assay (Fig. 4A) exhibited that NtMYB12 activated LUC activity in fluorescence imaging and in luciferase activity measurement (Fig. 4B-D) in tobacco leaf cells, 100-folds luciferase ratio of LUC/REN was significantly high in the cotransformed *oeNtMYB12* and *NtFLS* promoter relative to *NtFLS* promoter alone transform (Fig. 4B). 40-folds luciferase ratio of LUC/REN was significantly high in the cotransformed *oeNtMYB12* and *NtLAR* promoter relative to empty promoter alone (pGreen) transform (Fig. 4C). However, the luciferase ratio of LUC/REN did not significantly change in cotransformed *oeMYB12* and *NtDFR* promoter relative to empty promoter alone (Fig. 4D). It indicated that NtMYB12 physically activated *NtFLS* and *NtLAR* gene expression but did not affect *NtDFR* expression in tobacco leaf cells. Especially, when *NtFLS* promoter was disturbed to three fragments (Fig. 4E), NtMYB12 can also activate various MYB binding element regions driving *LUC* gene expression in tobacco leaf cells (Fig. 4F). Then, we performed above dual luciferase activity assay in vivo narcissus tepal protoplast. The same results were exhibited, except *oeNtMYB12* repressed *NtDFR* promoter driving *LUC* gene expression in narcissus tepal cells (Fig. 4G-H). Further, we detected NtMYB12 directly targeted and regulated *NtFLS*, *NtLAR* and *NtDFR* expression in vivo. The protoplasts of narcissus petal were prepared, collected, and transformed with the *oeMYB12-GFP* plasmid and alone GFP plasmid by PEG assay. Chromatin immunoprecipitation was performed with antibody against GFP, qPCR product was detectable in the promoter region of *NtFLS, NtLAR,* and *NtDFR* genes in IP. Calculation of the enrichment fold of IP relative to INPUT showed that NtMYB12 was significantly enriched in P1, P2, and P3 region of *NtFLS* gene and P2 region of *NtLAR* gene, as well as in the P1 region of *NtDFR* gene, but did not significantly changed in P1 region of *NtLAR* and P2 region of *NtDFR* gene. *ACTIN* gene was used as an inner control; GFP plant was used as an IP control (Fig. 4I-J). It indicates that NtMYB12 physically bound to the promoter of *NtFLS*, *NtDFR,* and *NtLAR* gene and activated *NtFLS,* and *NtLAR* expression but repressed *NtDFR* gene expression in flavonoid biosynthesis.

### NtMYB12 can interact with NtbHLH1 and NtWD40-1 to form MYB12-bHLH-WD40 (MBW) complex

**Fig. 5:**
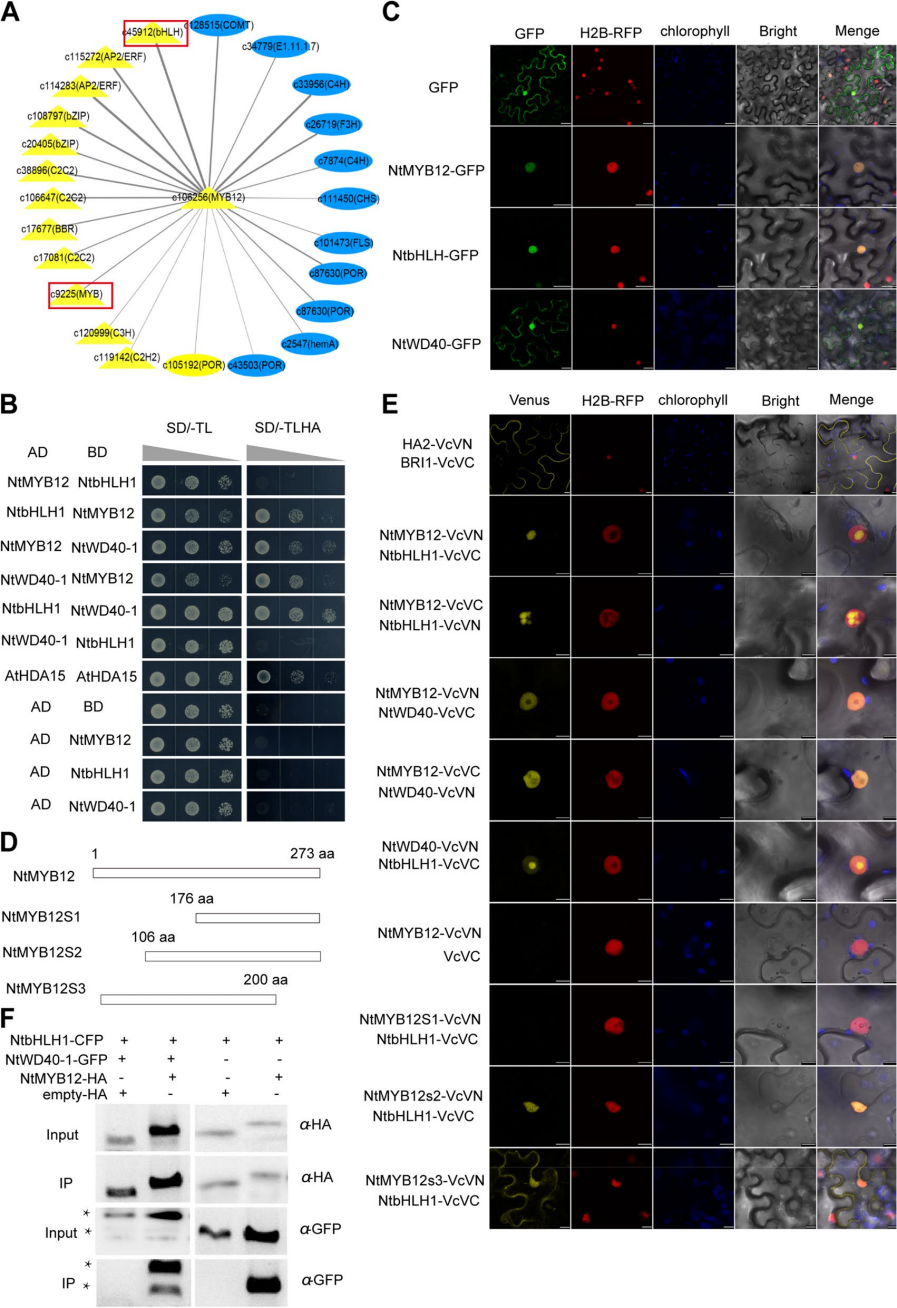
Identification of candidate proteins NtWD40-1 and NtbHLH1 of the MBW complex. **A** coexpression network of *NtMYB12* by WGCNA analysis; **B** NtMYB12 interacts with NtWD40-1 or NtbHLH-1 in yeast cells. Scale bars = 1 cm; **C** NtMYB12 colocalized with NtWD40-1 or NtbHLH-1 in the nucleus and cytoplasm of tobacco epidermal cells, Scale bars = 20 µm; **D** Scheme of various fragments of NtMYB12 used in bimolecular fluorescence complementation (BiFC) assay; **E** NtMYB12 interacts with NtWD40-1 or NtbHLH-1 in vivo by BiFC assay. Scale bars = 20 µm. **F** Coimmunoprecipitation detection of NtMYB12-HA with NtWD40-1-GFP and NtbHLH1-CFP or NtMYB12-HA with NtbHLH1-CFP by using antibody against HA and GFP for IP and immunodetection

NtMYB12 exists a conserved motif with a consensus sequence SG7 and SG7-2. This motif within the R3 domain has been suggested to play important role in interaction with the bHLH protein. To test whether, NtMYB12 still have possibility to interact with bHLH protein for their function although it has no typical bHLH binding sites. To this end, we firstly searched for the candidate members of the MYB12-bHLH-WD40 (MBW) complex. Based on the previous transcriptome dataset of *Narcissus tazetta* (NBCI, PRJNA750844, https://phytozome.jgi.doe.gov) (Yang et al. 2021), the coexpression network of *NtMYB12* was constructed by WGCNA, a common method for constructing gene co-expression networks (Peter and Steve 2008), several putative coregulators were exhibited (Fig. 5A). Of them, two candidate genes *NtWD40-1* and *NtbHLH1* were highly similar with the key regulatory members WD40 and bHLH of MBW complex in *Petunia* and *Arabidopsis.* The expression profile of three candidates during tepal development was detected by RT-qPCR and showed a similar expression profile (Supplementary Figure S5).

Next, we detected the interaction among the candidate proteins NtWD40-1, NtbHLH1, and NtMYB12 by yeast two hybrid and bimolecular fluorescence complementation assay. The results showed that yeast AH109 strain cotransformed plasmids *AD-NtMYB12* with *BD-NtWD40-1* or *BD-NtbHLH1* grew well in the medium (SD minus amino acids of LEU, ADE, TRP, and HIS), while colony cotransformed an empty AD vector with BD-NtWD40-1 or BD-NtbHLH1 did not grow there, suggesting that NtMYB12 can interact with NtWD40-1 in yeast cells (Fig. 5B). We further detected the localization of NtMYB12 and NtWD40-1 or NtbHLH1 in the nucleus and cytoplasm of the transformed tobacco leaf epidermal cells (Fig. 5C). Next, we confirmed the interaction of NtWD40-1, NtbHLH1, and NtMYB12 in vivo narcissus protoplast by BiFC. Cotransforming plasmids *NtMYB12-VcVN* with *NtWD40-1 VcVC* or *NtbHLH1- VcVC* with histone 2B fused RFP (H2B-RFP, positive control for nuclear localization) plasmid in narcissus protoplast, mVenus fluorescence signal was appeared in the nucleus (Fig. 5D). However, when the disturbed NtMYB12S1 without R3 domain was cotransformed with NtWD40-1 and NtbHLH1, Venus fluorescence signal was disappearing in the protoplast, while the disturbed NtMYB12S3 that deleted C3 domain of NtMYB12 was cotransformed with NtbHLH1, mVenus fluorescence signal was appeared in both the nucleus and cytoplasm. H2B-RFP was used as a control for transformation efficiency (Fig. 5E). Furthermore, coimmunoprecipitation of cotransformed tobacco leaf with *oeNtMYB12-HA*, *oeNtWD40-1-GFP*, *oeNtbHLH1-CFP* plasmids or with *oeNtMYB12-HA* and *oeNtbHLH1-CFP* plasmid using HA antibody showed that NtWD40-1 and NtbHLH1 or NtbHLH1 can been detected in IP by using GFP antibody, suggesting NtMYB12 physically interacted with NtWD40-1 and NtbHLH1 (Fig. 5F). These results indicate that NtMYB12 can interact with NtWD40-1 and NtbHLH-1 to form MBW triple complex via R3 domain.

### MBW specifically activated *NtDFR* gene expression in (pro)anthocyanin biosynthesis

**Fig. 6:**
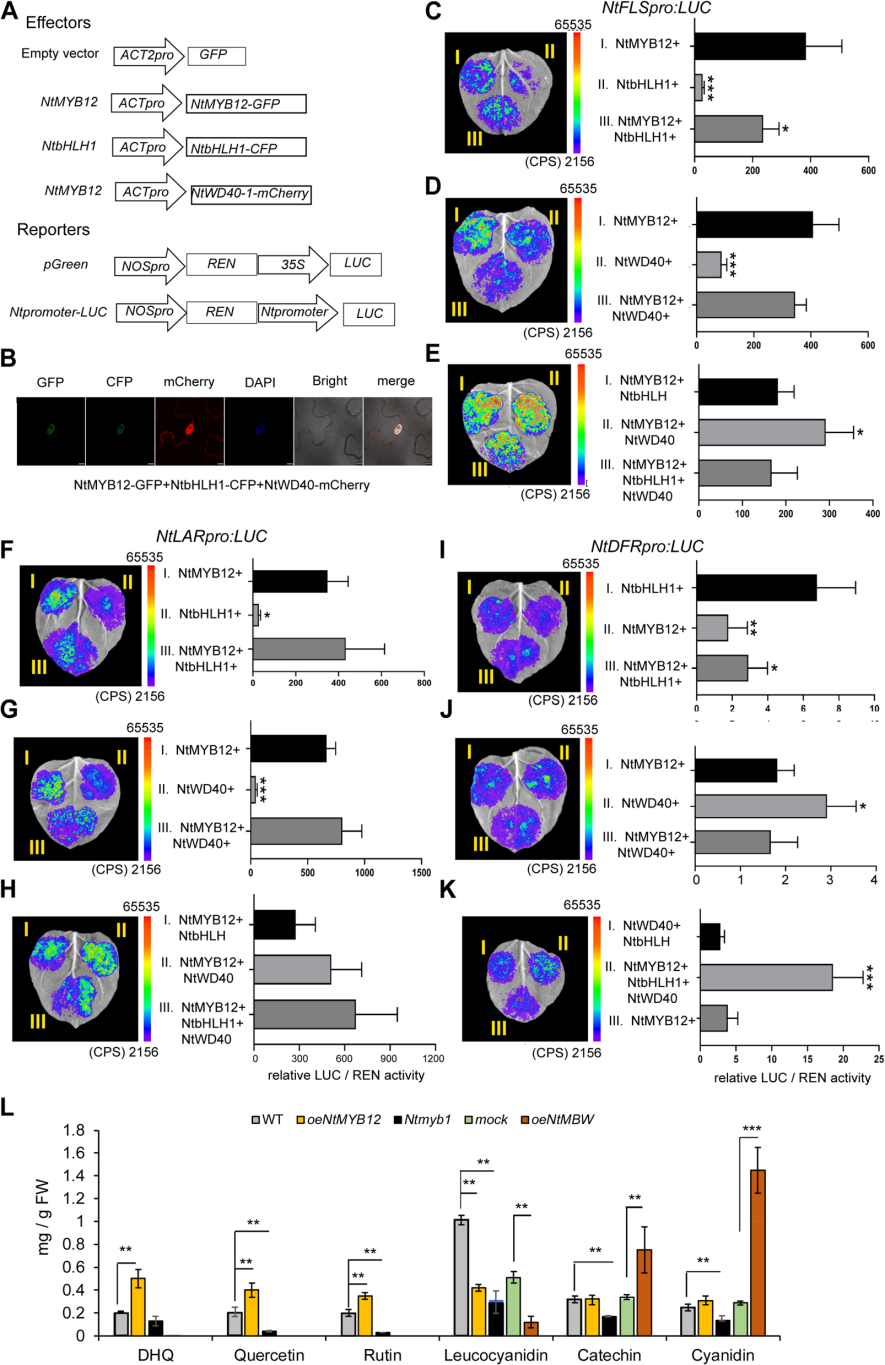
MBW specifically controls *NtDFR* expression, MYB12 alone activates *NtFLS* and *NtLAR* expression by dual-luciferase assay and the contents of flavonol, (pro)anthocyanin and anthocyanin in the *oeNtMYB12* and *Ntmyb12* tissue. **A** Scheme of effector and reporter; **B** The fluorescent imaging detected the protein expression of NtMYB12-GFP, NtWD40-1-mCherry and NtbHLH1-CFP in the cotransformed tobacco leaves under microscope. **C-E** luciferase fluorescent image and activity of cotransformed tobacco leaves with *oeNtMYB12-GFP/NtWD40-2-mCherry /NtbHLH1-CFP* and *NtFLSpro:LUC,* **F**–**H** and *NtLARpro:LUC,* (I)-(K) and *NtDFRpro:LUC* relative to empty vector, respectively; **L** the contents of flavonol, proanthocyanin, and anthocyanin in the *oeNtMYB12* and *Ntmyb12* narcissus callus tissue and in cotransformed narcissus leaves with *oeNtMYB12-NtWD40-1-NtbHLH1* relative to mock (WT). The error bar presented standard deviants of three biological replicates (*n* = 9). Asterisk presents the statistical significance compared to empty vector (C-K) or WT (L) (* *p* < 0.05; ** *p* < 0.01; ***p < 0.001)

In angiosperms, anthocyanin biosynthesis is regulated by conserved MYB/bHLH/WD40 (MBW) complexes, which formed by the interaction between MYB transcription factors, basic helix-loop-helix (bHLH) transcription factors, and WD40-repeat proteins (Ramsay et al., 2005; Xu et al. 2015). In *Narcissus*, whether MBW of MYB12-bHLH-1-WD40-1 is required for (pro)anthocyanin biosynthesis. To test this hypothesis, we detected the MBW activation on *NtFLS, NtDFR,* and *NtLAR* expression and metabolites by dual luciferase assay in tobacco leaf cells and narcissus petal protoplast (Fig. 6A). As above mention, NtMYB12 alone can activate *NtFLS* and *NtLAR* promoter driving *LUC* gene expression in narcissus and tobacco cells, but repressed *NtDFR* promoter driving LUC gene expression only in narcissus protoplast (Fig. 4F-G; Supplementary Fig. S6), when the combined plasmids of *NtMYB12-NtbHLH1, NtMYB12-NtWD40-1* or *NtMYB12-NtbHLH1-NtWD40-1* with *NtFLSpro-LUC, NtLARpro-LUC,* or *NtDFRpro-LUC* were co-injected in tobacco epidermal cells without promoter with LUC as negative control and 35S promoter with LUC as positive control (Supplementary Fig. S6), in which three fluorescence signals (NtMYB12-GFP, NtbHLH1-CFP and NtWD40-mCherry) were used as controls of three plasmid transformation efficiencies (Fig. 6B), the results showed that luciferase activity did not significantly change in the tobacco leaf cells coinjected by the plasmids of *NtMYB12-NtbHLH-NtWD40* with *NtFLSpro-LUC* or *NtLARpro-LUC* compared to alone NtMYB12 (Fig. 6C-H), however, surprisingly, luciferase activity increased significantly 10–100 folds in that coinjected by the combined plasmid of *NtMYB12-NtbHLH-NtWD40* and *NtDFRpro-LUC* or by the combined plasmids of *NtMYB12-NtbHLH1* or *NtMYB12-NtWD40-1* or *NtbHLH1-NtWD40-1* with *NtDFRpro-LUC* compared to NtMYB12 alone, respectively (Fig. 6I-K), MBW significantly promoted *NtDFRpro-LUC* signals, in MBW complex, it seems that both NtbHLH1 or NtWD40-1 promote the *NtDFR* promoter driving LUC expression (Fig. 6I-K), suggesting MBW specifically controlled *NtDFR* expression, MYB12 alone activated *NtFLS* and *NtLAR* expression and repressing *NtDFR* expression but both NtbHLH1 and NtWD40-1 alone activated *NtDFR* expression.

We then tested whether the elevated *MYB12* levels could lead to metabolite flux redirection in the *oeMYB12* line, enabling the Chinese narcissus to adapt to its particular color trait. The products of flavonoid and anthocyanin biosynthesis pathways were measured in the *oeNtMYB12* or the *Ntmyb12* and WT. Then, the contents of flavonol (Dihydroquercetin DHQ, Quercetin, Rutin), proanthocyanin (Leucocyanidin, Catechin), and anthocyanin (Cyanidin) in the *oeNtMYB12* and *Ntmyb12* tissue were measured and shown that the contents of flavonol were significantly accumulated in the *oeNtMYB12* tissue; in contrast, they were significantly declined in the *Ntmyb12* tissue (Fig. 6L). Interestingly, overexpressing *oeNtMYB12* alone did not significantly increase the contents of proanthocyanin (Leucocyanidin, Catechin) and anthocyanin (Cyanidin) in narcissus callus, but knockdown *Ntmyb12* significantly declined the contents of anthocyanin, suggesting that NtMYB12 was required for flavonol and (pro)anthocyanin biosynthesis. But NtMYB12 needs other regulators together to regulate anthocyanin biosynthesis.

To further test NtbHLH1 and NtWD40-1 with NtMYB12 (MBW) triplexes were involved in (pro)anthocyanin biosynthesis, we co-injected series of combinations of oe*NtbHLH1-NtWD40-1-NtMYB12* and oe*NtMYB12* alone to narcissus leaves (Fig. 6A), the products of flavonoid biosynthesis and anthocyanin biosynthesis pathways then were measured, the microscope checked the fluorescence of GFP, mCherry, and CFP for three plasmids successfully expressed (Fig. 6B). Apparently, the contents of flavonol and proanthocyanin were not significantly accumulated in the triplexes combinations relative to o*eNtMYB12* alone (Fig. 6L), however, that of leucocyanidin was significantly declined 6 folds while anthocyanin were significantly accumulated 2.5 folds in the triplex NtbHLH-NtWD40-NtMYB12 relative to *oeNtMYB12* alone (Fig. 6L) in narcissus leaves.

**Fig. 7:**
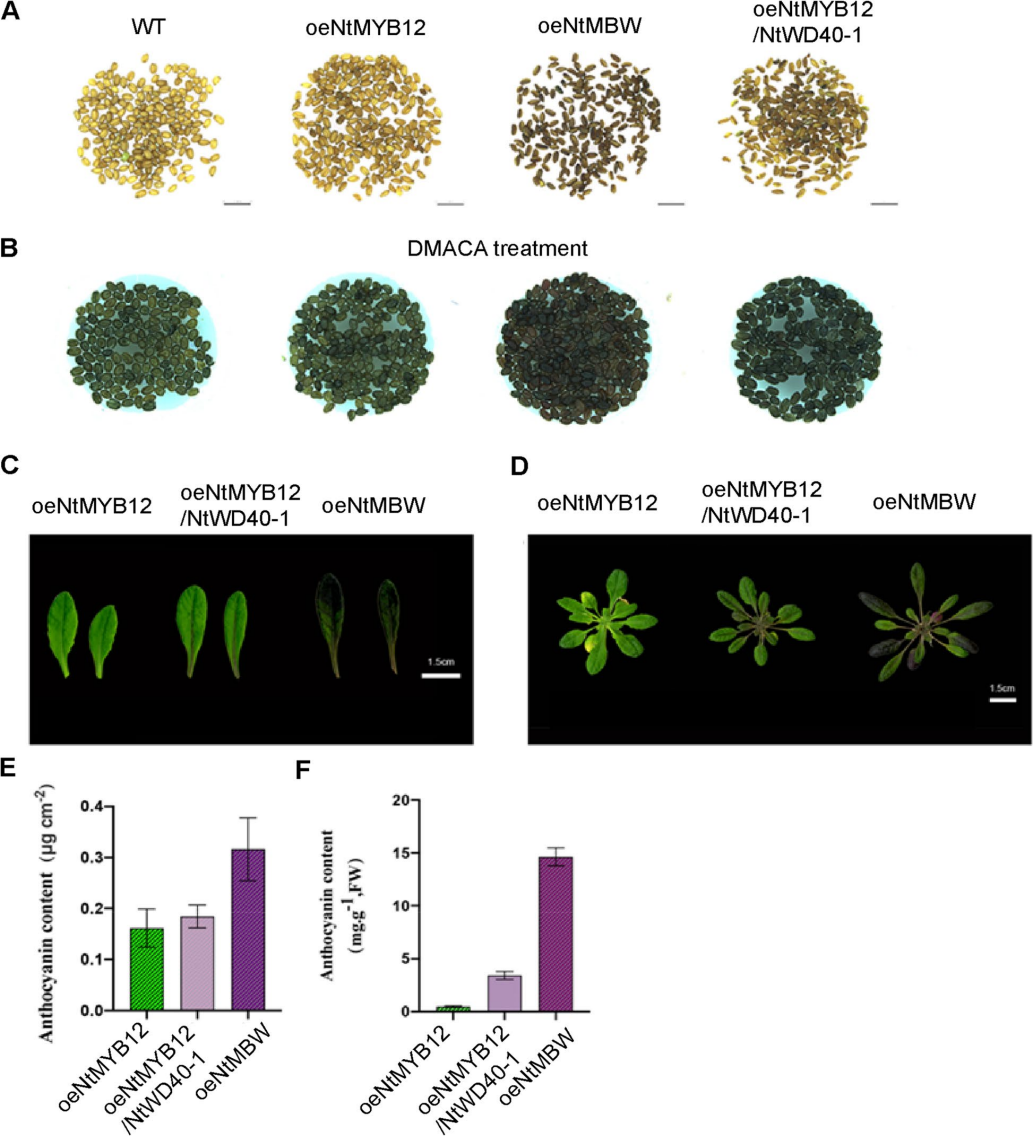
Phenotypic observations of oeNtMYB12 and oeNtMBW in *Arabidopsis*. **A-B** Observation on seed coat color of WT (Col-0), oeNtMYB12, oeNtMBW and oeNtMYB12/NtWD40-1plants before (**A**) and after DMACA staining (**B**). Sale bar = 1 cm; **C** Leaf scanning of 31st day after germination (DAG) old *oeNtMYB12*, *oeNtMBW* and *oeNtMYB12/NtWD40-1* plants; **D** Rosette scanning of 31 dag-old *oeNtMYB12*, *oeNtMBW* and *oeNtMYB12/NtWD40-1* plants; **E** The anthocyanin content of the 7th true leaf and 31 dag-old rosettes of *oeNtMYB12*, *oeNtMBW* and *oeNtMYB12/NtWD40-1* plants detected by portable Froce A and extraction method. Scale bar = 1 cm; **F** The anthocyanin content of t rosette leaf of 31dag-old *oeNtMYB12, oeNtMBW* and *oeNtMYB12/NtWD40-1* plants. The error bar presented standard deviants of three biological replicates (*n* = 12). Asterisk presents the statistical significance compared to *oeNtMYB12* plants (* *p* < 0.05; ** *p* < 0.01; ***p < 0.001)

The stable transformation of *oeNtbHLH-NtWD40-NtMYB12* in *Narcissus* and *Arabidopsis* was further performed to observe anthocyanin biosynthesis. Although *oeNtMYB12-NtbHLH-NtWD40* transformed narcissus fail to grow up, the *oeNtMYB12-NtbHLH-NtWD40* transformed *Arabidopsis* plants clearly exhibited a purple plant phenotype (Fig. 7A-D) and 15-fold high anthocyanin accumulation in leaves and twofold anthocyanin amount in seeds compared to the *oeNtMYB12* alone plants (Fig. 7E-F), indicating overexpressing *NtMYB12-NtbHLH-NtWD40* complex promote anthocyanin accumulation in *Arabidopsis*.

In addition, we further compared the coexpression network of NtbHLH1 and NtWD40-1 with NtMYB12 with metabolism profile of flavonoid biosynthesis and (pro)anthocyanin biosynthesis during tepal and corona development of *Narcissus tazett* and tepal and corona of two narcissus species *Narcissus tazett* and *Narcissus pseudonarcissu* species. Referring to previous data (Yang et al. 2021), interestingly, with the development of *Narcissus tazett* flower, the color of tepal was shifted from yellow to white, although the color of corona did not changed during development, the content of flavonol and proanthocyaynin were firstly upregulated at stage 1, then was immediately down-regulated (Yang et al. 2021), and the gene expression of *NtMYB12, NtbHLH1, NtWD40-1, NtFLS* and *NtLAR* were decline in both tepal and corona (Supplementary Figure S5), while yellow tepal and corona of *Narcissus pseudonarcissu* did not change during flower development (Supplementary Figure S7A), the content of flavonol and proanthocyaynin and the gene expression level of *NtMYB12, NtbHLH1, NtWD40-1, NtFLS, NtLAR* did not significantly change in both tepal and corona of *Narcissus pseudonarcissu* compared to *Narcissus tazett* (Supplementary Figure S7B-C). It further supported that NtMYB12-bHLH-WD40 complex requires for (pro)anthocyanin biosynthesis manipulating a colorized *Narcissus tazetta* “Jinzhanyintai” tepal.

## Discussion

**Fig. 8:**
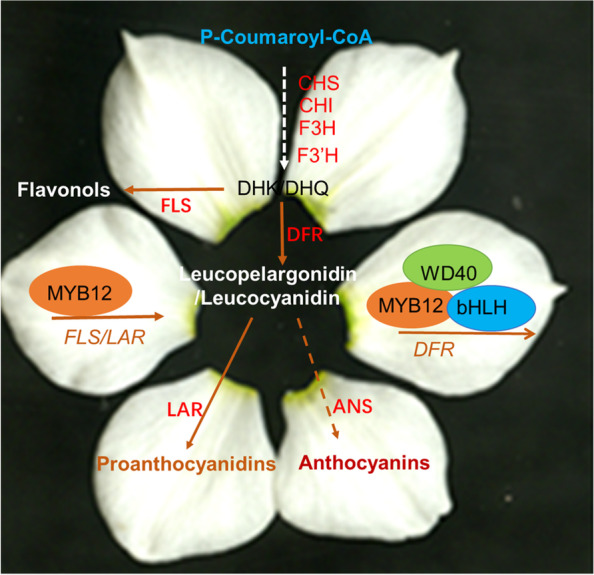
A working model for NtMYB12 or MBW determine flavonol or (pro)anthocyanin biosynthesis of narcissus petal. NtMYB12 dually functions on flavonol and proanthocyanin biogenesis via physically binding to *NtFLS* and *NtLAR* promoter activating their expression and on (pro)anthocyanin biosynthesis via NtMYB12-NtWD40-NtbHLH (MBW) triplex activating *NtDFR* and *NtANS* expression. Requirement of MBW complex for the competition between flavonol and anthocyanin biosynthesis results in narcissus colorized petal traits

In angiosperms, conserved MYB/bHLH/WD40 (MBW) complex regulates anthocyanin biosynthesis and acts in pyramidal fashion in a variety of model plants, including Arabidopsis, corn, petunia, snapdragon, alfalfa, etc. (Ramsay et al., 2005; Xu et al. 2015; Wang et al. 2019). While the MYB factor provides the DNA-binding specificity for the activation of the target genes, the other two components are often involved in regulating additional processes including different metabolites such as flavonol, proanthocyanin biosynthesis, at different tissue, at different developmental stage, which involves an MBW complex with the same bHLH transcription factor that regulates anthocyanin biosynthesis and a specific R2R3MYB protein that determines specificity for target genes. In narcissus, NtMYB12 is identified as a R2R3MYB protein, although it has no typical bHLH binding motif in R3 domain, it dually functions both on flavonol and proanthocyanin biogenesis via physically binding to the promoters of *NtFLS*, *NtLAR,* or *NtDFR,* activating *NtFLS*, *NtLAR* expression or repressing *NtDFR* expression and on anthocyanin biosynthesis via NtMYB12-NtbHLH1-NtWD40-1 triplex activating *NtDFR* expression, suggesting that requirement of NtMYB12-NtbHLH1-NtWD40-1 (MBW) complex for anthocyanin biosynthesis results in colorized narcissus petal traits. It provides a basis for manipulation of narcissus tepal colorization (Fig. 8).

In species evolutionary NtMYB12 is located between group and group, it contains R3 repeat, but has no typical bHLH protein-binding domain. The R3-MYB repressor contains only one R3 repeat. However, phylogenetic analysis shows that it can be further divided into two types: CPC-like and MYBL2-like, and the MYBL2-like type of R3-MYB repressor contains R3 repeat, and contains incomplete R2 repeats and is therefore more closely related to R2R3-MYB. NtMYB12 act not only as activator of *NtFLS* and *NtLAR*, but also as repressor of *NtDFR* in narcissus cells (Fig. 4; Figure S4), it can be explained by its protein primary structure, consistence with the facts like Arabidopsis AtMYB12 that contains a TLLLFR repressor motif at the carboxyl terminus, which is extremely important for its transcriptional repression function (Mehrtens et al. 2005; Dubos et al. 2008; Matsui et al. 2008; Stracke et al. 2010; Zhu et al. 2009), and is also similar to the MYB-like type of R2R3-MYB repressor, which is integrated into the MBW complex to inhibit floral pigment formation (Czemmel et al. 2009; Hichri et al. 2011; Wang et al. 2017; Shan et al. 2020). Furthermore, MYB protein is the core of the MBW complex, and the expression of its corresponding genes is spatiotemporally specific, so it determines that anthocyanins or proanthocyanidins are only accumulated in specific tissues in some special periods (Wang et al. 2020). Thus, it can explain overexpressing *NtMYB12* did not alter *NtDFR* transcript level in transformed narcissus callus, or *oeNtMYB12* alone did not change the expression level of LUC gene driven by *NtDFR* promoter in tobacco cells.

Numerous studies have shown that the amino-terminal R3 domain of MYB transcription factors that activate anthocyanin and proanthocyanidin biosynthesis contains a conserved motif that can be specifically recognized by the amino-terminal of R/B-like bHLH proteins—[D/E] Lx2[R/K]x3Lx6Lx3R (Zimmermann et al. 2004; Grotewold et al. 2000). Therefore, this type of MYB protein interact with members of bHLH protein subfamily IIIf, and WD40 to form an MBW complex and only MYB and bHLH proteins can directly interact with the promoters of downstream genes sequence binding (Ma and Constable, 2019). Different MBW complexes regulate anthocyanin and procyanidin biosynthesis in a tissue-specific manner. While bHLH and WD40 are often constitutively expressed, affecting the accumulation of anthocyanin or proanthocyanin in plants, it also plays a role in other aspects, such as petunia's AN1, which encodes a bHLH protein, it affects vacuolar pH and seed coat morphology (Sheehan et al. 2012; Butelli et al. 2019). Corresponding to most dicots, in the monocot maize, both the early and late anthocyanin biosynthesis genes appear to be transcriptionally regulated by the MBW complex (Mol et al. 1998). In Chinese narcissus, an extremely low transcript level of *NtDFR* and *NtANS* in late stage of tepal might associate with the level of *NtMYB12, NtWD40-1* and *NtbHLH* was declined paralleledly. Although NtMYB12 did not change *NtDFRpro* driving *LUC* gene expression in vitro tobacco cell, when overexpressing *NtMYB12-NtbHLH-1-NtWD40-1* significantly activates *NtDFRpro* driving *LUC* gene expression in tobacco leaves (Fig. 6) and promotes anthocyanin accumulation in *Arabidopsis* and *Narcissus* leaves (Figure S7). Although NtMYB12 can interact with NtWD40-1 and NtbHLH1 to form a MBW complex to control *NtDFR* expression in *Arabidopsis* and *Narcissus*, NtMYB12 is a repressor in proanthocyanin or anthocyanin biosynthesis, NtWD40-1 and NtbHLH1 might specifically act activators for NtDFR homeostasis in proanthocyanin or anthocyanin biosynthesis, since NtWD40-1 and NtbHLH1 activated *NtDFR* expression while NtMYB12 repressed *NtDFR* expression (Fig. 6). *NtANS* expression level might be affected by other unknown additional regulator for anthocyanin biosynthesis.

Many different types of transcription factors such as MYB, bHLH, WD40, WRKY, Zinc finger, and MADS transcription factors are involved in the regulation of flavonoid biosynthesis (Terrier et al. 2009; Yang et al. 2021), but most widely studies are on MYB transcription factors and MYB-bHLH-WD40 (MBW) complex. The MYB transcription factor in the huge majority of dicotyledonous plants prefer directly regulating universal genes and early biosynthesis genes of phenylpropane/flavonoid biosynthesis and the MBW ternary complex with MYB as the core regulates the late biosynthetic genes of flavonoids (anthocyanins/proanthocyanins) in a variety of model plants, including Arabidopsis, corn, petunia, snapdragon, alfalfa, etc. (Hichri et al., 2011; Wang et al. 2019). In MBW triple complex, NtWD40-1 and NtbHLH1 might compete with NtMYB12 targeting to the promoter of NtDFR, thus MBW acts as activator for *NtDFR* expression, which is consistence with that facts that high level of MYB96 in *Arabidopsis* was a repressor in ABA signaling but low level of MYB96 interacting with HDA15 and chromatin remodeling BRM complex, work as activator for ABA induced response, in a MYB96 protein dose dependent manner (Lee and Seo 2019; Lee et al. 2015). Narcissus NtMYB12 function as a "restrictive factor" regulating flavonol, proanthocyanidin or anthocyanin biosynthesis in MBW ternary complexes, respectively, leading to colorless tepal in *Narcissus tazetta* in evolutionary manner. It is worth noting that NtMYB12 seems to be a pioneer factor for upstream of phenylpropane/flavonoid biosynthesis (Fig. 2, Figure S3), and the tepal colorless formation in narcissus was competed by metabolite flux between phenylpropane affected by fragrance formation and flavonoid biosynthesis (Yang et al. 2021). The detail mechanism will be need to further address.

## Methods

### Materials

*Narcissus tazetta* cultivated variety “Jinzhanyintai” material was grown and collected as the description in previous research (Ren et al. 2017; Yang et al. 2021). Tepals were first collected at the fifth day (sample S1) as sheathed leaves emerged from the bulbs, then collected at another three stages (samples S2–S4) as umbel in spathe developed. All samples were immediately frozen in liquid nitrogen and stored at -80^0^C.

The transgenic callus material of *Narcissus tazetta* was constructed on the basis of the stable genetic transformation system of narcissus callus established previously (Patent CN202210162166.8). Briefly, narcissus ovary in S0 ~ S1 period was used as explants to induce callus after disinfection, and then Agrobacterium EHA105 carrying NtMYB12 plant overexpression vector (*35Spro:NtMYB12-GFP-GUS*) and *NtMYB12-RNAi* vector were used to transform narcissus callus after 3 days of co-cultivation, it was transferred to the screening medium containing 25 μg/mL kanamycin. After 27 days of screening, it was used for material identification and experimental analysis.

Tobacco *Nicotiana benthamiana* was planted in a long-day climate chamber. The long-day culture conditions were a photoperiod of 16 h/8 h, a temperature of 22 °C, and a humidity of 60%) was used for transient injection experiments.

#### Plasmid construction

The construction of *NtMYB12-RNAi* vector refers to the method of Pu et al. (2012). Construction of *35Spro:NtMYB12-GFP* vector was used the *pK7FWG2* vector as the backbone, the *35Spro:NtMYB12-GFP* vector was constructed in two steps by GATEWAY cloning. Briefly, the full-length cDNA sequence of *NtMYB12* (stop codon removed), was cloned to *pENTR* vector (Invitrogen) by homologous recombination with the D-TOPO® gatewayvector and sequenced correctly, The LR reaction was performed with TOPO-NtMYB12 and the expression vector *pK7FWG2* to obtain the final vector *pK7FWG2-NtMYB12* (*35Spro:NtMYB12-GFP-GUS*). Other plasmids were obtained by using Novozyme's "ClonExpress® II One Step Cloning Kit" (C112) kit to obtain the linearized vector and the target gene insert by homologous recombination with the adapter sequence in infusion cloning the manner. *pCBIM-GFP-Flag* (*35S:GFP*) was used as a positive control in narcissus protoplast transformation experiments; *pK7FWG2* (*35S: GFP*) and modified *pCAMBIA3301* (*ACT2pro:GFP*) were used for plant overexpression vectors. *pRNAi-GG* (Accession No. JQ085427.1) was used for the construction of the NtMYB12-RNAi vector. *pGreen* (*T7pro:LUC*) used for the construction of the dual luciferase vector was driven by the T7 promoter firefly luciferase gene (LUC)) expression as a reporter system, and Renilla luciferase gene (REN) expression driven by NOS promoter as an internal standard. *pGADT7* (AD) and *pGBKT*7 (BD) were used for the construction of yeast two-hybrid vectors. *pRTVcVN* (Accession No. MH373677) 0.1, referred to as *VcVN*; *pRTVcVC* (accession number MH373678.1) (He et al. 2018), *VcVC*; *pRTVnVN* (referred to as *VnVN*) and *pRTVnVC* (referred to as *VnVC*) for bimolecular fluorescence complementation experiments (BiFC), where *pRTVnVN* and *pRTVnVC* as a negative control.

#### Genome walking assay

According to Takara's Genome Walking Kit (Code No.6108) manual, it is designed to clone the upstream sequences of *NtFLS, NtLAR, NtDFR* and *NtANS* CDs 5' end, respectively, and the primer sequences are shown in the Supplementary Table S2.

#### Protoplast isolation and transformation

The isolation of *Narcissus tazetta* leaf and tepal cell protoplasts referred to the isolation methods of Arabidopsis and rice protoplasts (Zhang et al. 2011), with slight modifications. The tepals from 4–5 flowers at development stage 4 incubated and shacked in fresh enzyme solution at 60 rpm speed under dark condition for 4 h. After the solution became feculent, the same volume of W5 buffer was added, then followed the above method (Zhang et al. 2011) to collect the protoplasts. For the transformation of *Narcissus tazetta* leaf and tepal cell protoplasts, refer to the method of Yoo et al. (2007) and Zhang et al. (2011).

#### Bimolecular fluorescence complementation assay

The *VcVN-NtMYB12, VcVN-NtbHLH1, VcVN-NtWD40-1, VcVC-NtMYB12, VcVC-NtbHLH1, VcVC-NtWD40-1* plasmids were extracted by the "EndoFree Plasmid Midi Kit" (CW2105), then were co-transformed to the protoplasts of *Narcissus tazetta* mesophyll cells and observed the fluorescence under a laser confocal microscope (Leica SP8) after 16 h. Co-transfection of VnVN and VnVC empty vector was used as the negative control.

#### Dual luciferase imaging and activity measurement

The dual luciferase experiment refers to the method of Rodriguez (2004), with slight changes. The constructed *ACT2pro:NtMYB12-GFP, ACT2pro:NtWD40-1-mCherry, ACT2pro:NtbHLH1-CFP* overexpression vector (as effector), *NtFLSpro:LUC*, *NtLARpro:LUC, NtDFRpro:LUC* vector (as reporter), GFP-tagged empty vector (as negative control for effector) and *pGreen* empty vector (as reporter) The positive control) was transferred into Agrobacterium GV3101 (pSoup-p19) competent cells, and the positive clones were screened and identified, and the Agrobacterium resuspended solution with various combinations of effector and reporter was mixed in an equal ratio of 1:1 and used for tobacco injection. the experimental group (e.g. *ACT2pro:NtMYB12-GFP* + *NtFLSpro:LUC*), the negative control (GFP empty + *NtFLSpro:LUC*) and the positive control group (*pGreen*) should be injected into the same leaf. The injected tobacco plants were cultured in the dark for 12–24 h. After 2–3 days, the GFP fluorescence signal was observed under a fluorescence microscope or a laser confocal microscope to illustrate the expression of effector protein.

The luciferase activity of the samples was qualitatively analyzed by using the plant in vivo imaging system (the Immunization Center of Fujian Agriculture and Forestry University). Subsequently, the relative luciferase activity of the samples was quantified using a microplate reader according to Huang et al. 2020. The fluorescence signal value of firefly luciferase/renilla luciferase (LUC/REN) was used to represent the relative luciferase activity.

#### Co-immunoprecipitation (Co-IP) and immunoblotting assays

The combination of constructs expressing the corresponding MYB12-HA-tagged proteins and NtbHLH1-CFP or NtWD40-1-GFP-tagged proteins were co-transformed into narcissus protoplasts. After incubation 16 h, the transfected protoplasts were harvested for the Co-IP and immunoblotting assays as described previously (Zheng et al. 2018). The HA-tagged proteins were immunoprecipitated with anti-HA antibody coupled beads (anti-HA affinity matrix, Roche). The immunoprecipitated proteins were then separated via 13.5% SDS-PAGE, blotted and analyzed by immunoblotting with anti-GFP (TransGen, HT801, 1:1,000 dilution) and Anti-HA-Peroxidase (Roche, 3F10, 1:500 dilution) antibodies.

#### ChIP-qPCR

According to the description of Huang et al. (2020). A little modification was as follow: The narcissus petal protoplasts transformed with oeNtMYB12-GFP and oeGFP plasmid were collected and isolated the chromatin, and the cross-linked DNA fragments ranging from 200 to 1000 bp in length were immunoprecipitated by an antibody against the GFP (TransGen, HT801). The enrichment of the selected promoter regions of target genes was determined by comparing the amounts in the precipitated and non-precipitated (input) DNA samples, by quantitative PCR using designed region-specific primers (Supplementary Table S1). The experiments were performed in triplicate. *NtACTIN* were used as controls in qPCR.

#### Measurement of flavonoids and anthocyanin metabolites by HPLC analysis

Tepals, cornas from S1 to S4 stages of *Narcissus tazetta* and transformed callus tissue, as well transformed Arabidopsis plants were collected for analysis the contents of flavonoids and (pro)anthocyanin. HPLC were performed to analysis the extracts of flavonoids metabolism according to the description by our previous publication (Ren et al. 2017; Yang et al. 2021). The flavonoids and anthocyanin standards including naringenin, dihydroquercetin, kaempferol, quercetin, lutein, Leucocyanidin, Catechin, and Cyanidin were purchased from Sigma-Aldrich (St. Louis, MO, USA), rutin and zeaxanthin were purchased from TCI (TCI Co., Ltd, Japan) and Sangon (Shanghai, China), respectively. All flavonoids and (pro)anthocyanin standards were prepared in MeOH/DMSO (1:1, v/v) and MTBE (containing 0.01% BHT), respectively, and stored at -20 °C before use.

#### Illumina RNA-seq library construction and transcriptome sequencing

The total RNA of *Narcissus tazetta* was extracted using the protocol described by Ren et al. (2017).

The Illumina RNA-seq library can be constructed with mRNA isolated from *oeNtMYB12* and *Ntmyb12* tissue. Transcriptomes were sequenced using the Illumina HiSeqTM- 2500 platform for high-throughput sequencing of short reads RNA seq. Clean data were obtained by removing reads containing adapters, reads containing poly-N, and low-quality reads. These clean reads were then mapped to the reference transcriptome sequence using software (Riechmann et al. 2000). Only reads with a perfect match or one mismatch were further analyzed and annotated based on the reference transcriptome. The mapped fragments for each gene were counted by feature Counts and fragments per kilobase per million mapped reads (FPKM) were calculated. Differential expression analysis between two samples was performed using the EBSeq R package (Ogata et al. 1996). The resulting *P* values were adjusted using the Benja-mini and Hochberg’s approach for controlling the false discovery rate (Jia et al. 2004). Genes identified by EBSeq with FDR 0.01, FC >  = 2, were defined as differentially expressed.

#### Bioinformatics analysis of transcriptome sequencing data

Illumina RNA seq needs to assemble the offline data, Sequence assembly of high-quality sequencing data using Trinity software (Trapnell et al. 2010; Sharon et al. 2013). The detail procedure was reference to manual of Biomarker technologies company.

#### WGCNA analysis

WGCNA is a common method for constructing gene co-expression networks (Peter and Steve 2008). Through WGCNA analysis was according to the description by Yang et al. (2021).

#### Data statistical analysis methods

The new Duncan's multiple range test in one-way ANOVA was used; The Kruskal–Wallis test in the unpaired test was used when they were not uniform. The different significance represented the different letters. And *p* < 0.05 means a significant difference.

### Supplementary Information

**Additional file 1: Fig. S1.** Alignment of NtMYB12 with different R2R3 MYB proteins of Sg7 clade and its evolutionary position. **Fig. S2.** Genome wide transcriptome sequencing and analyze their reprograming transcriptome in gain/loss of *NtMYB12* transgenic tissue compared to wildtype. **Fig. S3.** Up- and downregulated genes of phenylpropanoid biosynthesis and flavonoid biosynthesis pathways in the *oeNtMYB12*, WT, and *Ntmyb12* lines by matching heatmap and MapMan metabolite pathway. **Fig. S4.** The expression levels of *NtDFR* and *NtANS* in narcissus petal protoplast transformed transiently by *oeNtMYB12* and *Ntmyb12* plasmids relative to WT. **Fig. S5.** The expression profiles of three candidates NtMYB12, NtbHLH1, and NtWD40-1 during tepal and corona development were detected by RT-qPCR. **Fig. S6.** NtMYB12 activates *NtFLS* and *NtLAR* expression and represses NtDFR expression by dual-luciferase assay (controls for Fig. 6). **Fig. S7.** NtMYB12 and related downstream gene expression profiles and pigment contents in *Narcissus tazetta* and *Narcissus pseudonarcissus* species. **Table S1.** The list of primer pairs used in this study. **Table S2.** The promoter sequences of *NtFLS,* *NtLAR* and *NtDFR* contain many MYB-motives.

**Additional file 2: Dataset S1.** The differentially expression genes of *oeNtMYB12* relative to WT (FC >2, *P* value <0.01).

**Additional file 3: Dataset S2.** The differentially expression genes of *Ntmyb12* relative to WT (FC >2, *P* value <0.01).

## Data Availability

All raw data were deposited in the GenBank NCBI Short Read Archive (https://www.ncbi.nlm.nih.gov/sra/PRJNA891931). The materials provided are contacted to Ying Miao (ymiao2013@hotmail.com, or ymiao@fafu.edu.cn).

## Abbreviations

GPPS: Geranyl diphosphate synthase
PAL: Phenylalanine ammonia-lyase
C4H: Cinnamic acid 4-hydroxylase
4CL: 4-Coumarate: CoA ligase
HCT: Shikimate O-hydroxycinnamoyltransferase
C3'H: 5-O-(4-coumaroyl)-D-quinate 3'-monooxygenase
CCoAOMT: Caffeoyl-CoA O-methyltransferase
CCR: Cinnamoyl-CoA reductase
CAD: Cinnamyl-alcohol dehydrogenase
F5H: Ferulate-5-hydroxylase
CHS: Chalcone synthase
CHI: Chalcone isomerase
F3H: Flavanone 3-dioxygenase
F3'H: Flavonoid 3'-monooxygenase
FLS: Flavonol synthase
DFR: Dihydroflavonol 4-reductase
LAR: Leucoanthocyanidin reductase
ANS: Anthocyanidin synthase
CoIP: Coimmunoprecipitation
ChIP: Chromatin remodeling
BiFC: Bimolecular Fluoresence Complementation
MBW: MYB-bHLH-WD40 triplex
LUC: Luciferase
