# Supplementary material for: NtMYB12 requires for competition between flavonol and (pro)anthocyanin biosynthesis in Narcissus tazetta tepals
Source: Mol Hortic. 2023 Feb 8;3:2. doi: 10.1186/s43897-023-00050-7 (PMC10515073; doi:10.1186/s43897-023-00050-7)
Supplement: Supplementary file 1 — Additional file 1: Fig. S1. Alignment of NtMYB12 with different R2R3 MYB proteins of Sg7 clade and its evolutionary position. Fig. S2. Genome wide transcriptome sequencing and analyze their reprograming transcriptome in gain/loss of NtMYB12 transgenic tissue compared to wildtype. Fig. S3. Up- and downregulated genes of phenylpropanoid biosynthesis and flavonoid biosynthesis pathways in the oeNtMYB12, WT, and Ntmyb12 lines by matching heatmap and MapMan metabolite pathway. Fig. S4. The expression levels of NtDFR and NtANS in narcissus petal protoplast transformed transiently by oeNtMYB12 and Ntmyb12 plasmids relative to WT. Fig. S5. The expression profiles of three candidates NtMYB12, NtbHLH1, and NtWD40-1 during tepal and corona development were detected by RT-qPCR. Fig. S6. NtMYB12 activates NtFLS and NtLAR expression and represses NtDFR expression by dual-luciferase assay (controls for Fig. 6). Fig. S7. NtMYB12 and related downstream gene expression profiles and pigment contents in Narcissus tazetta and Narcissus pseudonarcissus species. Table S1. The list of primer pairs used in this study. Table S2. The promoter sequences of NtFLS, NtLAR and NtDFR contain many MYB-motives. [file 43897_2023_50_MOESM1_ESM.pdf]

Yang et al., NtMYB12-bHLH-WD40 complex requires for competition between flavonol and proanthocyanin biosynthesis in *Narcissus tazetta* tepals

Supplementary Figures and the legends

A

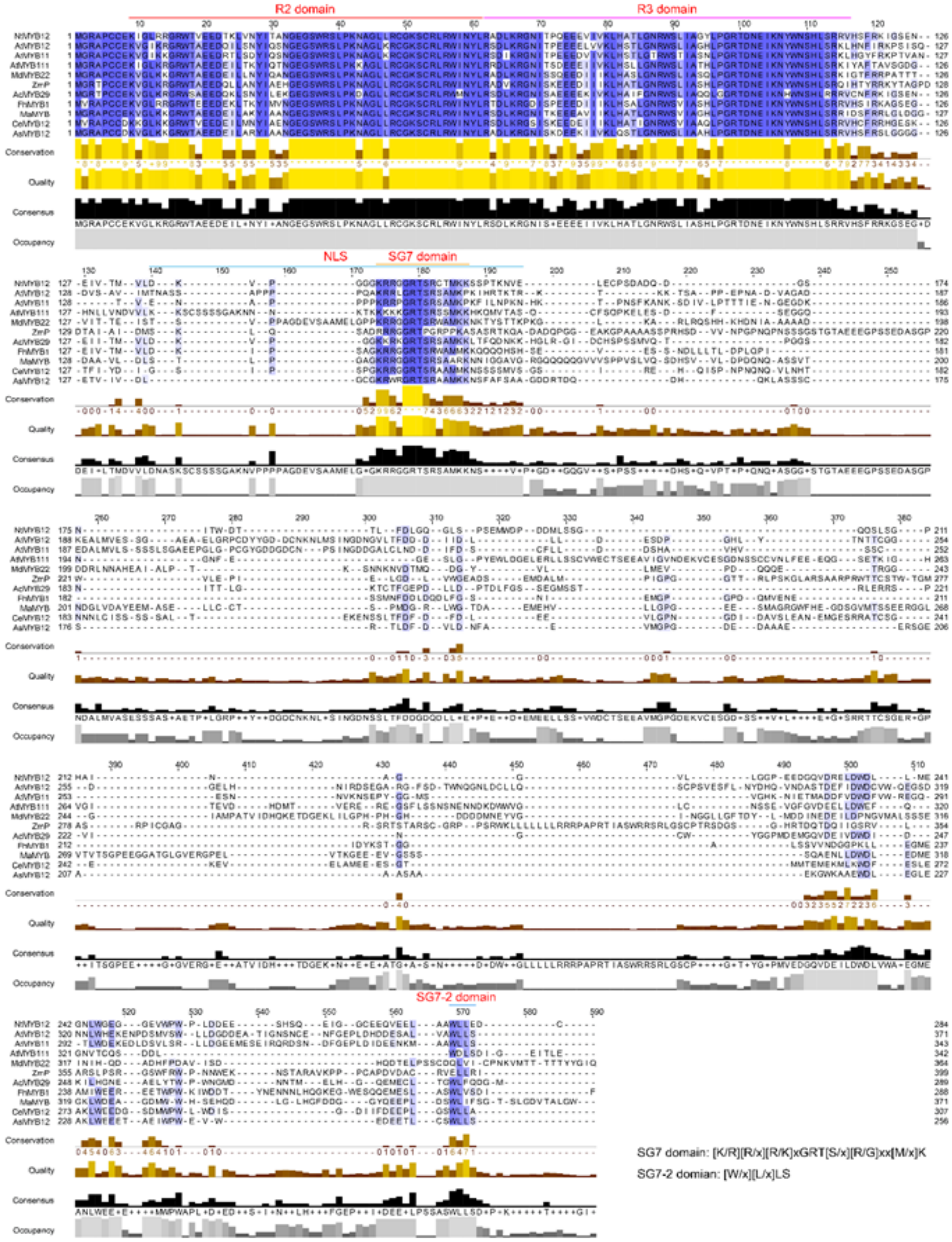

B

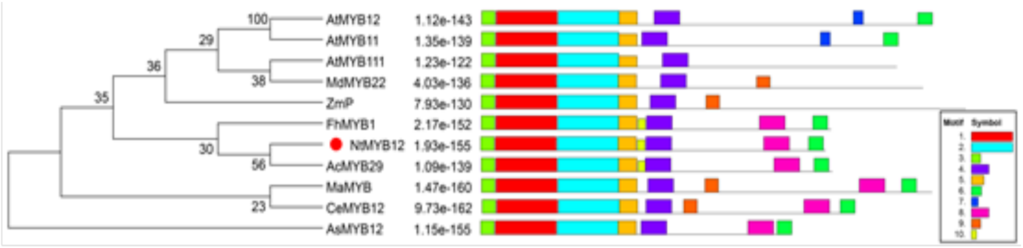

Supplementary Figure S1 Alignment of NtMYB12 with different R2R3 MYB proteins of Sg7 clade and its evolutionary position.

(A) Alignment of NtMYB12 with different R2R3 MYB proteins of Sg7 clade; **(B)** The evolutionary position of NtMYB12. Motif 1, R2 domain; Motif 2, NLS domain; Motif 3, SG7 domain; Motif 4, R3 domain; Motif 6, SG7-2 domain.

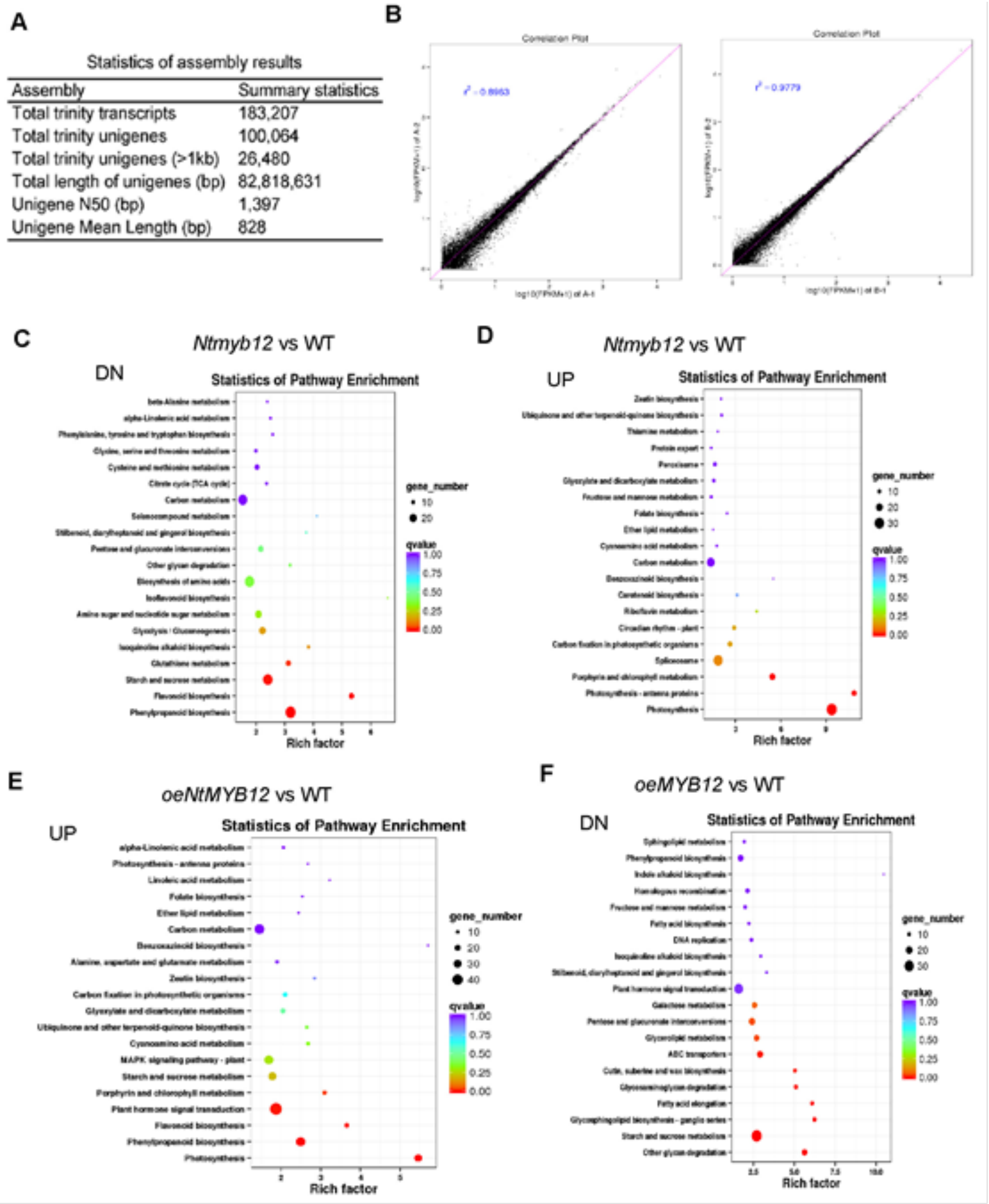

Supplementary Figure S2 Genome wide transcriptome sequencing and analyze their reprograming transcriptome in gain/loss of *NtMYB12* transgenic tissue compared to wildtype.

(A) Total collected unigenes (>1kb) from *NtMYB12* transgenic tissue; **(B)** The variants of three replicates (r2=0,97) *Ntmyb12* or *oeNtMYB12* relative to WT; **(C)-(F)** Plots exhibited

KEGG enriched pathway of DEGs of *Ntmyb12* or *oeNtMYB12* relative to WT (IFCI>=2, P value <0.01). C downregulated pathway in *Ntmyb12* relative to WT, D upregulated pathway in *Ntmyb12* relative to WT, E upregulated pathway in *oeNtMYB12* relative to WT, F downregulated pathway in *oeNtMYB12* relative to WT.

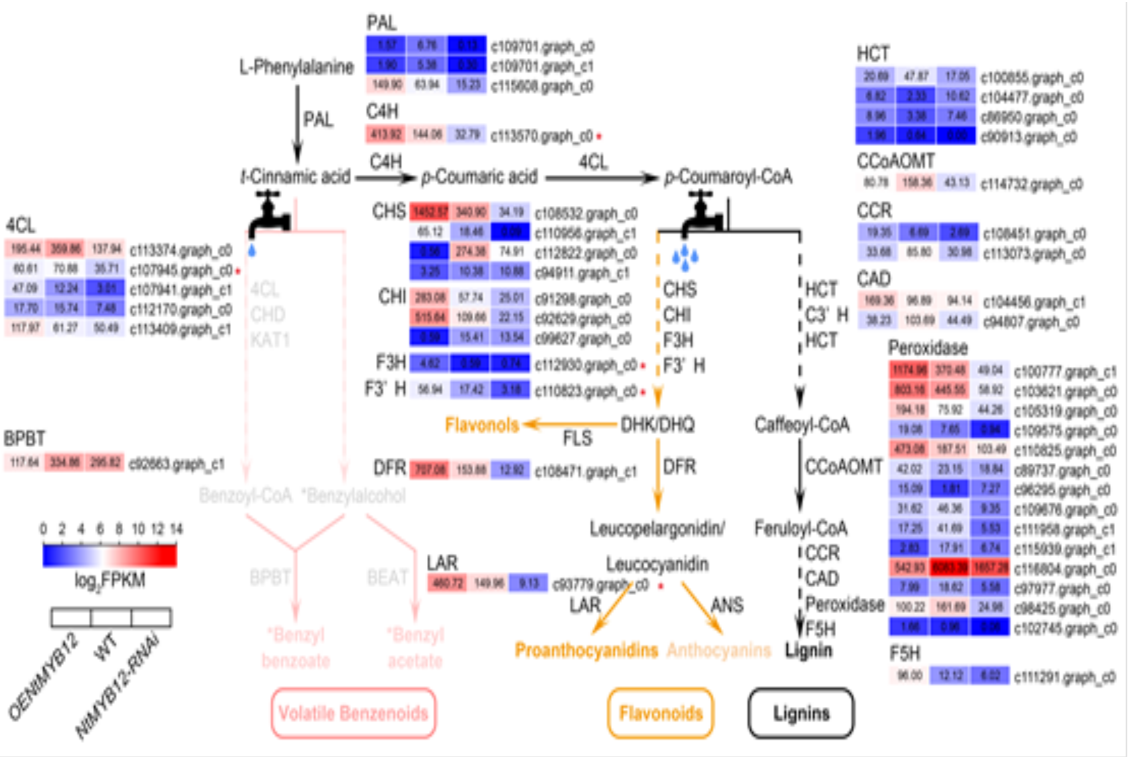

Supplementary Figure S3 Up- and downregulated genes of phenylpropanoid biosynthesis and flavonoid biosynthesis pathways in the *oeNtMYB12*, WT, and *Ntmyb12* lines by matching heatmap and MapMan metabolite pathway.

Red, upregulated; blue, downregulated.

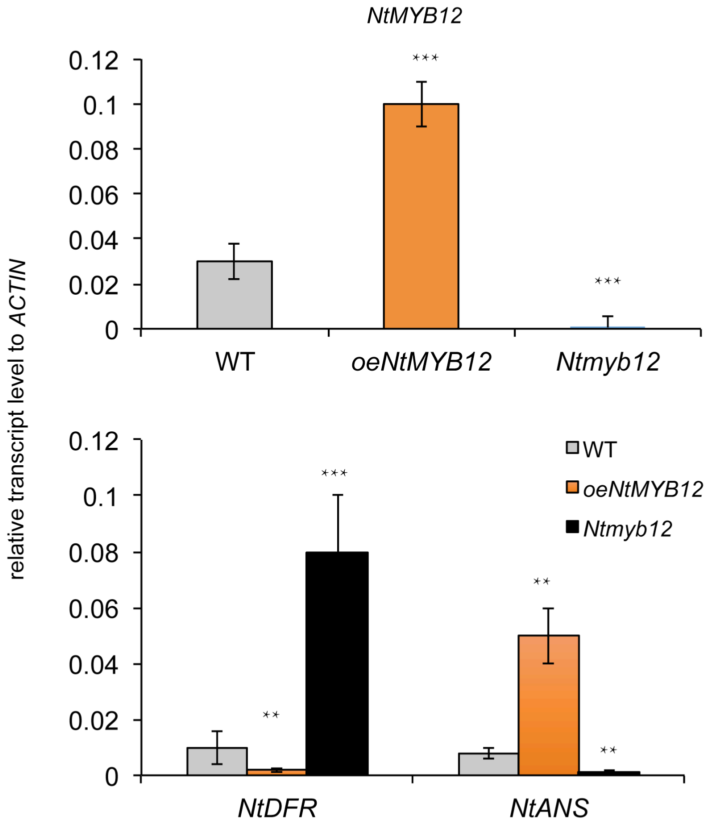

Supplementary Figure S4 The expression level of *NtDFR* and *NtANS* in the narcissus petal protoplasts transformed by *oeNtMYB12* and *Ntmyb12* plasmids compared to WT. The expression levels of *NtMYB12, NtDFR, NtANS*. The error bar presented standard deviants of three biological replicates. Asterisk presents the statistical significance compared to T1 (** p<0.01; ***p<0.001).

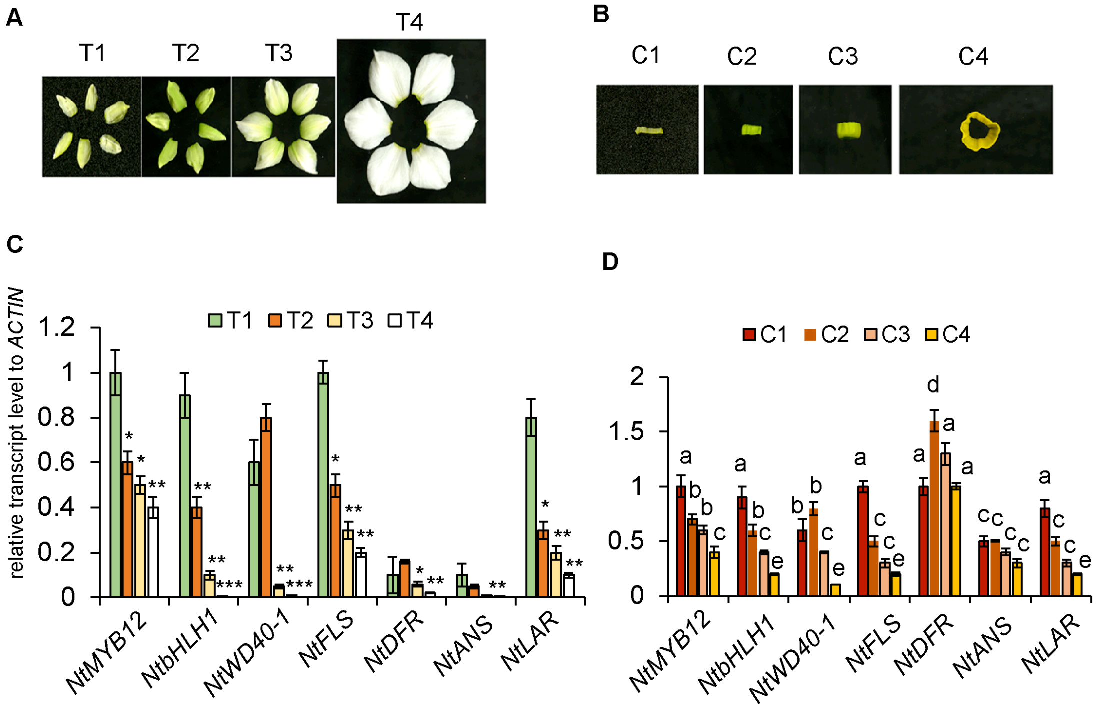

Supplementary Figure S5 The expression profile of three candidates during tepal and corona development was detected by RT-qPCR.

(A) The tepal during development of Narcissus tazetta; (B) The corona during development of Narcissus tazetta; (C)-(D) The expression levels of NtMYB12, NtWD40, NtbHLH, NtFLS, NtLAR, NtDFR, NtANS during tepal (C) and corona (D) development. The error bar presented standard deviants of three biological replicates. Asterisk presents the statistical significance compared to T1 (* $p<0.05$; ** $p<0.01$; ***$p<0.001$). The new Duncan's multiple range test in one-way ANOVA was used, different letter presents the significant different ($p < 0.05$).

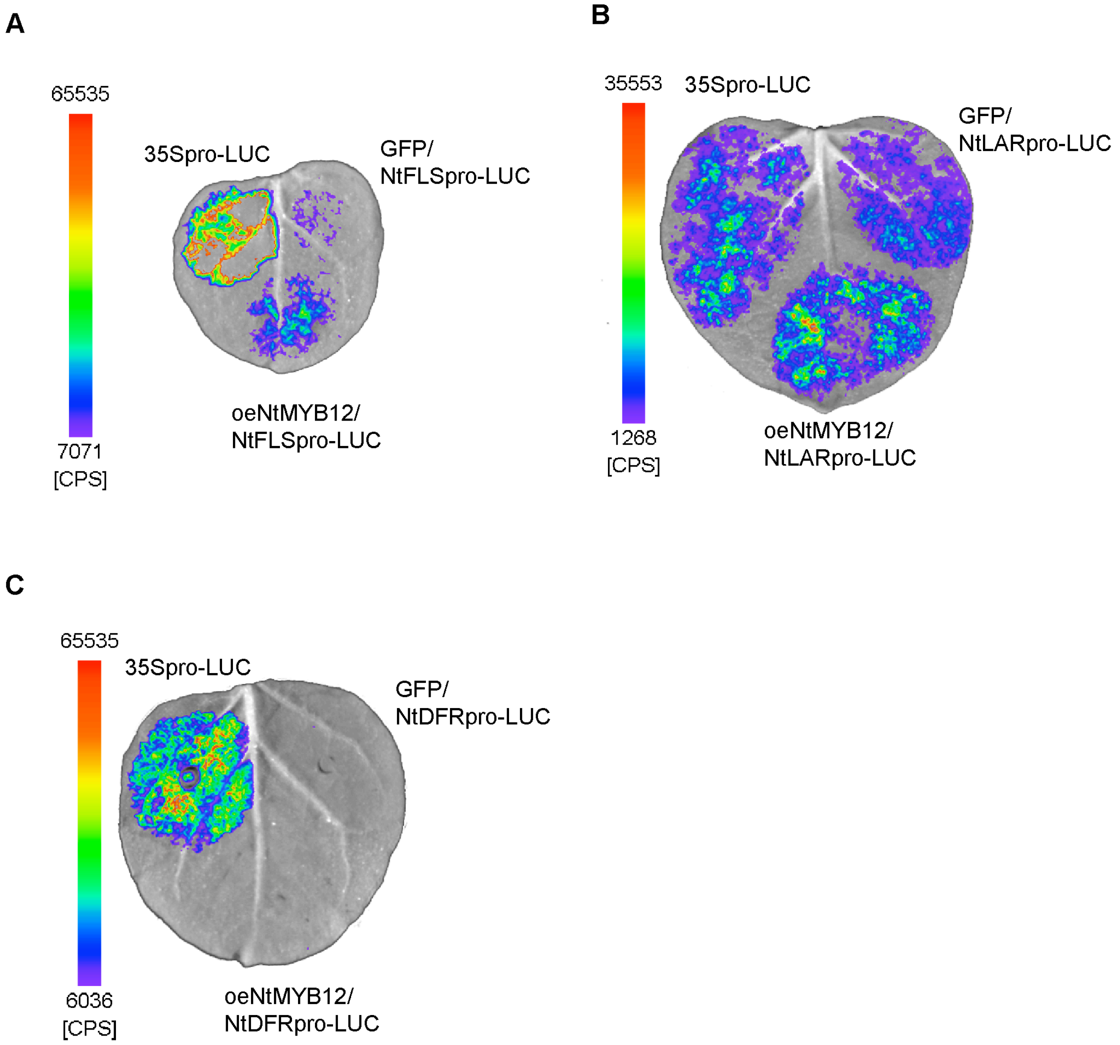

Supplementary Figure S6 NtMYB12 activates *NtFLS* and *NtLAR* expression by dual-luciferase assay (controls for Figure 6)

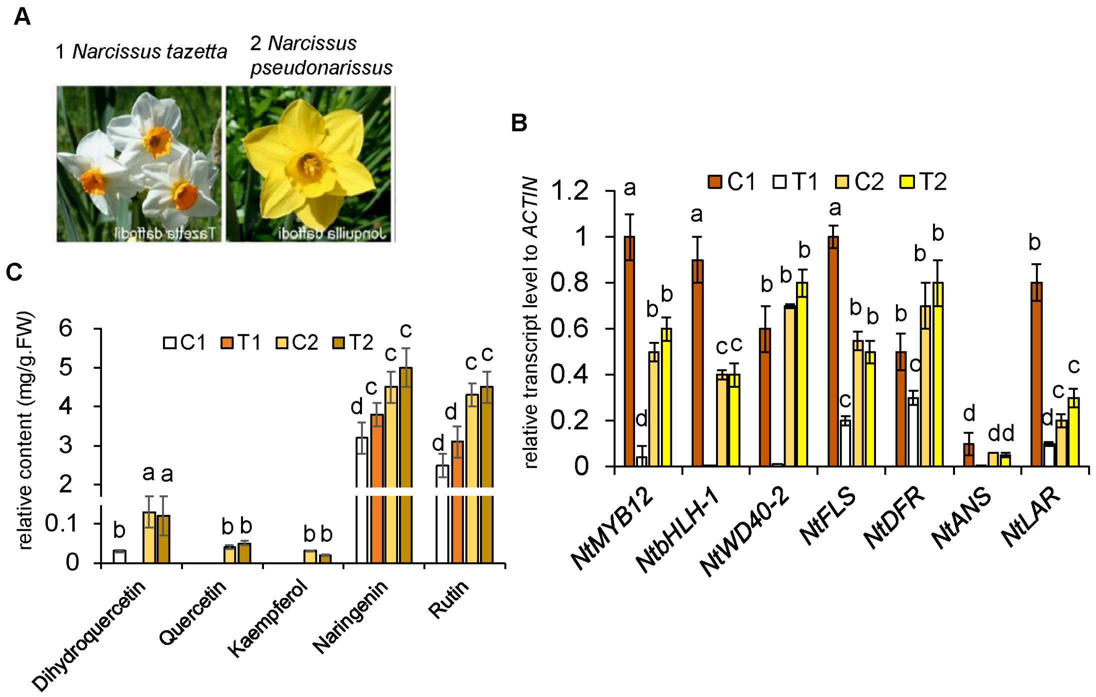

Supplementary Figure S7 *NtMYB12* expression profile and pigment contents in *Narcissus tazetta* and *Narcissus pseudonarcissus*.

(A) The corona and tepal of two species at T3 stage; **(B)**The expression levels of Nt*MYB12, NtWD40, NtbHLH, NtFLS, NtLAR, NtDFR, NtANS* in two species of narcissus. **(C)** The content of Rutin and (pro)anthocyanin in corona and tepal of two species; The error bar presented standard deviants of three biological replicates. the new Duncan's multiple range test in one-way ANOVA was used, different letter presents the significant different ($p < 0.05$).

Supplementary Table S1 The list of primer pairs in this study

qPCR:

NtActin_F c72145/f1p4/2737 GAGAGGGGTTACTCATTCAC

NtActin_R CTCATAGCTCTTCTCCACTG

NtMYB12_F c106256.graph_c0 CCGAAGAATGCAGGTTTGTT

NtMYB12_R AACGACCACCTGTTTCCAAG

NtFLS_F c101473.graph_c0 AGAAGGATCTGGAAGGGAAG

NtFLS_R GATACGCAGTAGGTGTTTGG

NtDFR_F c33974/f1p6/1644 TAAGTTCCAAGACGCTATCC

NtDFR_R GAATGACATCTCCTCCAACA

NtANS_F c121090.graph_c1 CATGGAAGAGAAGGTGAAGC

NtANS_R CCTGCAAGAGATAGGAAGTG

NtLAR_F c28704.graph_c0 GAGTCACCATAACTGAGCAG

NtLAR_R CGTCTTGTATAAGCTGTCCG

Plasmid constructs

AD-NtMYB12_F ggccagtgaattccacccgggATGGGAAGGGCTCCATGTTG

AD-NtMYB12_R attcatctgcagctcgagctcTTAACAATCTTCAAGCAGCCAAGC

AD-NtbHLH1_F ggccagtgaattccacccgggATGGCGGAGAGACGGAGGAGGAA

AD-NtbHLH1_R attcatctgcagctcgagctcTCACATTGCATTTTGGAAGCCAGC

AD-NtWD40-1_F ggccagtgaattccacccgggATGGAATTTTTAGAGGGGAACTTCG

AD-NtWD40-1_R attcatctgcagctcgagctcTTAATGTGATGCATCTAGCAGTTGC

BD-NtMYB12_F atggccatggaggccgaattcATGGGAAGGGCTCCATGTTG

BD-NtMYB12_R ccgctgcaggtcgacggatccTTAACAATCTTCAAGCAGCCAAGC

BD-NtbHLH1_F atggccatggaggccgaattcATGGCGGAGAGACGGAGGAGGAA

BD-NtbHLH1_R ccgctgcaggtcgacggatccTCACATTGCATTTTGGAAGCCAGC

BD-NtWD40-1_F atggccatggaggccgaattcATGGAATTTTTAGAGGGGAACTTCG

BD-NtWD40-1_R ccgctgcaggtcgacggatccTTAATGTGATGCATCTAGCAGTTGC

VcVN/C-NtMYB12_F tatccagatccagtgggatccATGGGAAGGGCTCCATGTTGTG

VcVN/C-NtMYB12_R cgcactagtaagcttggtaccACAATCTTCAAGCAGCCAAGCAG

VcVN/C-NtbHLH1_F tatccagatccagtgggatccATGGCGGAGAGACGGAGGAGGAA

VcVN/C-NtbHLH1_R cgcactagtaagcttggtaccCATTGCATTTTGGAAGCCAGC

VcVN/C-NtWD40-1_F tatccagatccagtgggatccATGGAATTTTTAGAGGGGAACTTCG

VcVN/C-NtWD40-1_R cgcactagtaagcttggtaccATGTGATGCATCTAGCAGTTGC

ACT2pro: NtMYB12_F gtgtgtgacctcgagactagtATGGGAAGGGCTCCATGTTGTG

ACT2pro: NtMYB12_R ataccaggtggaggtcccccgggACAATCTTCAAGCAGCCAAGCAG

35Spro: NtMYB12_F caccATGGGAAGGGCTCCATGTTGTG

35Spro: NtMYB12_F ACAATCTTCAAGCAGCCAAGCAG

NtMYB12_RNAi_F accaggtctcaggagGACCAGAAGAGGACGGACAA

NtMYB12_RNAi_R accaggtctcatcgtTGACAAGCACCCTTTAACAAT

NtDFRpro_F ctatagggcgaattgggtaccGGGGCTTAGGAGTATTATCGTCAT

NtDFRpro_R gcatggcgactgcaggaattcCATTGTCGGATTTTCGAAGAAAA

NtDFRpro-LUC_F GAAAATCCGACAATGgaattcctcgagatggaagacgccaa

NtDFRpro-LUC_R	gcatggcgactgcaggaattcttacacggcgatctttccgc
pGreen_F	cgaagtcatgtcgacctcgagttgagagtgaatatgagact
pGreen_BamHI_R	tttggcgtcttccatctcgagGGATCCgtcctctccaaatgaaatga
NtFLSpro1-920_F	ctatagggcgaattgggtaccAGCAGAGATTGTCTAGTTAT
NtFLSpro1-600_F	ctatagggcgaattgggtaccGATTCCCTTGTTTTTGAAGG
NtFLSpro1-280_F	ctatagggcgaattgggtaccTTTCCTCCGCTTTTCGATCC
NtFLSpro_R	gtcttccatctcgagGGATCCTGTTGATTCTTTGCGAGGGG
NtLARpro_F	ctatagggcgaattgggtaccGGGAATGTAGAGGGAAATGTGG
NtLARpro_R	gtcttccatctcgagGGATCCCTTTTCTTTTCTCTTCTCCT
NtMYB12-GFP_F	gtgtgtgacctcgagactagtATGGGAAGGGCTCCATGTTGTG
NtMYB12-GFP_R	gtccttgtagtccatactagtACAATCTTCAAGCAGCCAAGCAG
NtbHLH1-GFP/CFP_F	gtgtgtgacctcgagactagtATGGCGGAGAGACGGAGGAGGAA
NtbHLH1-GFP/CFP_R	gtccttgtagtccatactagtCATTGCATTTTGGAAGCCAGC
NtWD40-2-GFP_F	gtgtgtgacctcgagactagtATGGAATTTTTAGAGGGGAACTTCG
NtWD40-2-GFP_R	gtccttgtagtccatactagtATGTGATGCATCTAGCAGTTGC
NtbHLH1-mCherry_F	cctgcaggcggccgcactagtATGGCGGAGAGACGGAGGAGGAA
NtbHLH1-mCherry_R	CTCGCCCTTGCTCACggcgcgcccCATTGCATTTTGGAAGCCAGC
NtWD40-1-mCherry_F	cctgcaggcggccgcactagtATGGAATTTTTAGAGGGGAACTTCG
NtWD40-1-mCherry_R	CTCGCCCTTGCTCACggcgcgcccATGTGATGCATCTAGCAGTTGC

Supplementary Table S2 The sequences of NtMYB12 and the promoter sequences of *NtFLS, NtLAR, NtDFR*

>NtMYB12_c106256.graph_c0
ATGGGAAGGGCTCCATGTTGTGAGAAAATCGGGTTGAGGAGAGGAAGGTGGACTGTAGAGGA
GGATACGAAGTTGGTTAACTACATTACGGCCAATGGTGAAGGTTCCTGGAGATCTTTGCCGAAG
AATGCAGGTTTGTTGAGATGCGGAAAGAGCTGCAGGCTGCGTTGGATAAACTACTTGAGAGCA
GACCTGAAGAGAGGTAACATTACTCCGCAGGAAGAGGAGGTGATAGTAAAGCTACATGCAACG
CTTGGAAACAGGTGGTCGTTAATAGCGGGGTATCTTCCAGGGAGAACAGATAACGAGATCAAG
AACTACTGGAATTCCCACCTCAGCAGGAGGGTGCACAGCTTCCGCAAGATCGGGAGCGAAAAC
GAGATCGTCACGATGGTCCTGGACAAGGTTCCCGGTGGGGGCAAGCGGCGTGGCGGCCGAAC
GAGCCGGTGCACCATGAAGAAGAGCTCCCCGACCAAGAACGTGGAGCTCGAATGCCCGAGTG
ATGCCGATCAGGACGGAAGCAACATAACCTGGGACACCACATTGTTTGACCTGGGTCAAGGCT
TGTCGCCGAGTGAGATGTGGGACCCAGATGACATGCTCAGCTCTGGTCAGCAGAGCTTGAGTG
GGCCCCATGCAATCAATGCCGGTGGGGTGCTGCTGGGGGGACCAGAAGAGGACGGACAAGTT
GACCGTGAACTAGATTGGGACCTGTTGATGGAGGGAAATTTATGGGGCGAGGGTGGGGAGGTG
TGGCCATGGCCACTGGATGACGAGGAGTCGCATTCCCAAGAAATTGGAGGGTGCGAAGAGCA
AGTGGAAGAGTTGGCTGCTTGGCTGCTTGAAGATTGTTAA

>NtFLSpro1-920_MH472580.1
AGCAGAGATTGTCTAGTTATATATTAGTATAGATATATTAGTATAGATGAATTCTCTATTGATTAA
AAAATACCGAACTCTACGACAATATGACAGTATATGTAGTACTAGGATACAAAGTTCTCCTAGC
ACTAATGGACTGGGAGTTGGGACAAACCCCACGCAGACGAACCCTTTAAGGGGCCGTTTGGAG
AAGGTTTTTTAGACCGGTTTTCTACTTTTTTTACTGTTTCAACAGTAAAAACAAGGGAAAGATAAA
AACCCTATTTGGCAAAATACTTGGCTTTTTCAATTCCTATTTGGCAAAAAACCGGTTTTTTGATTC
CCTTGTTTTTGAAGGGATTTTGAGGGTTTTTACTATTCATCCGTACTTTCTCTCTATCTCTCTCCTC
CATATCACCAAATTTCACCACATTCCATCTTCCTCTTTTATTCTCAAACAAGATTATCCAATACCC
ATATTAAAAAAACCCCACTTTTGCCAAACAAGTTTTTTTGCATTTTTTAATTTTTCTTACACGGTTT
TTTGAATCTCAGTTTTTTTGCCAAACGGGCCCTAAGAGTTTGGTCACTTGGTCCAACATATTTGCG
TGTCTCGTCTGCCACTTCTTTTATTATCATTTTTTTTGTTTATTTTTTTTTCCTCCGCTTTTCGATCCA
TCATGAAAAAAGCACCGCAGATTACAAGGGACCAGTCCCATGTGGCTTTTGATCTAGTTTCAAA
CCGGTGGAGATGAGTGAATTTCAGCCTTACTTCCCGCTGACTTCATTCATGGTTGGTAGTTGCAA
CAAGTGACCTTAGAACCCCCACGTAGTTCGTGTGTTTGTTACCCACGTCTCCCCCCTCCTACTTA
TACCCCACACCAAGTGTTCTCCTAACTATCTCACTTTACTCTCCTCCCCTCGCAAAGAATCAACA

>NtFLSpro1-600
GATTCCCTTGTTTTTGAAGGGATTTTGAGGGTTTTTACTATTCATCCGTACTTTCTCTCTATCTCTC
TCCTCCATATCACCAAATTTCACCACATTCCATCTTCCTCTTTTATTCTCAAACAAGATTATCCAAT
ACCCATATTAAAAAAACCCCACTTTTGCCAAACAAGTTTTTTTGCATTTTTTAATTTTTCTTACACG
GTTTTTTGAATCTCAGTTTTTTTGCCAAACGGGCCCTAAGAGTTTGGTCACTTGGTCCAACATATT
TGCGTGTCTCGTCTGCCACTTCTTTTATTATCATTTTTTTTGTTTATTTTTTTTTCCTCCGCTTTTCGA
TCCATCATGAAAAAAGCACCGCAGATTACAAGGGACCAGTCCCATGTGGCTTTTGATCTAGTTT
CAAACCGGTGGAGATGAGTGAATTTCAGCCTTACTTCCCGCTGACTTCATTCATGGTTGGTAGTT
GCAACAAGTGACCTTAGAACCCCCACGTAGTTCGTGTGTTTGTTACCCACGTCTCCCCCCTCCTA

CTTATACCCCACACCAAGTGTTCTCCTAACTATCTCACTTTACTCTCCTCCCCTCGCAAAGAATCA
ACA

>NtFLSpro1-280
TTCCTCCGCTTTTCGATCCATCATGAAAAAAGCACCGCAGATTACAAGGGACCAGTCCCATGTG
GCTTTTGATCTAGTTTCAAACCGGTGGAGATGAGTGAATTTCAGCCTTACTTCCCGCTGACTTCA
TTCATGGTTGGTAGTTGCAACAAGTGACCTTAGAACCCCCACGTAGTTCGTGTGTTTGTTACCCA
CGTCTCCCCCCTCCTACTTATACCCCACACCAAGTGTTCTCCTAACTATCTCACTTTACTCTCCTC
CCCTCGCAAAGAATCAACA

>NtLARpro_MH371155.1
GGGAATGTAGAGGGAAATGTGGTGAAATGTGGTGATGTGAAGGAGAGAGAGACAACCGTAGG
GTGAACAGTAATAACGAAAAAATCTGTGAAAATTCTTTCGAAATGAAGGGAATCCAAGGGAAG
GGGATAACGCAGTGAATTCCACCTGAGCCAAACCTGTTATCGAATTTCACCTGCAAATCTACTGT
AACTACAGTAATAACAAGCCCGCTTTTCGCAAATAACGGGTCTCCCAAATAGCCCTAAGGGGAC
AGTAAAGTTAGAAAGAGAGATAATTATGCGTAGGTCAATACTATTACAGGGCTGCAGATAAATA
ATAAATTATTGTTAGAGGATTTAATTTAAGACTTGACTTTTCAAAGAATAAAAAATTGAATCACTG
CAGCTACTGTTAAAACTAATATGTTGCATTTAAAATAGGCTAAATCTCACTTTACTCTCCTAAACT
ATAGGCTGTTTCATCGTTCAGTACACAAATCTTTTAAATTACCAGTATGCCCACCTGAATCTGATA
TTCTCACCCCCTATTTTTGACACGAAAAAAGGAAAAGGGTACAAGTGGGTTGACAATATAAGAA
TAAAATGAAAAATAAGGGGTGAGAATATCAGATTCAGAGGGCATAGTGTTAATTTAAAAGATTT
GAATACTAAATGGTGAAAACGCATATAGTTTAGAGGGACAAAATGAGATTTAGTCTTTAAAATAT
CAGGTGGCTCAATATGAGATTATAAATCATGTCGTTTTCTTCATATTGGAAAAAAAAAAAAAAAA
ATTTGAAAAAATATATATCATCCAAACAACTTCGTTCGTGATGAGTCCATACAGCTACCCCGTTC
AAAGACACGTATGGACTTCAAGAACTGAGAAGGTAGTTGGCCAAGGCCAACCAACTTATATATA
AAGAGAAGGCCACACTCCCATTTTCATCAAGCAGAGAATAAGGGATTGATAATTTTGTGGAGGA
AATTAAGAAAGGAGAAGAGAAAAGAAAAG

>NtDFRpro_MH367491.1
GGGGCTTAGGAGTATTATCGTCATTTGACAGGGTAATTTTGAATTTTCGGTGAAATTACAAAATA
ATTCGAATTTGGGGTTGTTTTATGAAATACGATAGATTTGGGGTCCAATTTTGAATTAATCCTTCT
TTTTATCTCGAGCCCTAGACCCACTCCGTGATGCCTATCCCCATCCGTTGCCTCCCCGTGCTGCC
CACCTCCTGATCGTCGCCTCCCTGTGCCACCCATCCCTCGGCCAGCTCCTCCTCTAGAGCCACC
CCTCTCCCGTGGCCATCACCTCCTGCAGCCGCCTCTAATTCCGCTTCCCCTCCTGCGGTCGTTCC
CGTCCCTCTACCTCCTCTAGCCCGAAGACGATGGCCTCATGGACAAGGACACTGTCGGCGACC
CATCCCCTATTGGTCCGCCCGTGCCCTAGCTGAGATCTACCATGACCAGTGGCGACATGGCGAG
GAGGTAATGGCACCAGTCAAAAGAAGAGGATTGATATCAACTTTTAATGGTAATTTCTTGTCCTT
CAATTAGATTTCATTCAGTTGATTGTCAGCAAATTTTTCAGATCCAAAATTTGTATGCATTAATTC
CAACTAATTTGAGAATTTTGCAAATGTTCTGTTTTGAATTTTTTTGGATTATGTCTACAAGTTGTTC
GATCAAATGTTTCAACATTAGGACCTGGCGACACACATCTGGGTGGTGATGATTTTGACAAGGT
CTGTTGAAACTCCTCTTTTAAATAACAGTGAAATAGTTAGAAAAAAATCAATGGGAAAATAATTC
AAGGGAAAAAAAATCCAGTTTACATAGACCAGTCAAAAATTGTTAGATGAGAACACATGCTTGA
AGGGTTAGGTGAAAGCAAGAACATTAGTATAAATATGATGTGCTCCTTCTCAATTATGTACAAAT
ATTTCCTGCTTAAATTGCTTTCTTGTTATTTTTTTCTTCGAAAATCCGACAATG
